# Author Correction: Tau binding protein CAPON induces tau aggregation and neurodegeneration

**DOI:** 10.1038/s41467-019-10990-8

**Published:** 2019-07-01

**Authors:** Shoko Hashimoto, Yukio Matsuba, Naoko Kamano, Naomi Mihira, Naruhiko Sahara, Jiro Takano, Shin-ichi Muramatsu, Takaomi C. Saido, Takashi Saito

**Affiliations:** 1 Laboratory for Proteolytic Neuroscience, RIKEN Center for Brain Science, 2-1 Hirosawa, Wako-City, Saitama, 351-0198 Japan; 2 Department of Functional Brain Imaging Research, National Institute of Radiological Sciences, National Institutes for Quantum and Radiological Science and Technology, 4-9-1 Anagawa, Inage-ku, Chiba-City, Chiba, 263-8555 Japan; 3 Division of Neurology, Jichi Medical University, 3311-1 Yakushiji, Shimotsuke-City, Tochigi, 329-0498 Japan; 4 Center for Gene & Cell Therapy, The Institute of Medical Science, The University of Tokyo, Tokyo, 108-8639 Japan; 5 Department of Neuroscience and Pathobiology, Research Institute of Environmental Medicine, Nagoya University, Nagoya-City, Aichi 464-8601 Japan

**Keywords:** Cell death in the nervous system, Alzheimer's disease

Correction to: *Nature Communications* 10.1038/s41467-019-10278-x, published online 03 June 2019.

The original version of this Article contained an error in Fig. 2b, in which the representative image for ‘*App*^*NL-G-F*^’ mice at 24 months (bottom row, far right) was inadvertently duplicated from the image to its left, the representative image for the ‘WT’. This has been corrected in both the PDF and HTML versions of the Article.

