# Supplementary material for: Tau binding protein CAPON induces tau aggregation and neurodegeneration
Source: Nat Commun. 2019 Jun 3;10:2394. doi: 10.1038/s41467-019-10278-x (PMC6546774; doi:10.1038/s41467-019-10278-x)
Supplement: Supplementary file 1 — Supplementary Information [file 41467_2019_10278_MOESM1_ESM.pdf]

Supplementary information

Tau binding protein CAPON induces tau aggregation and neurodegeneration

Hashimoto et al

Supplementary Figure 1

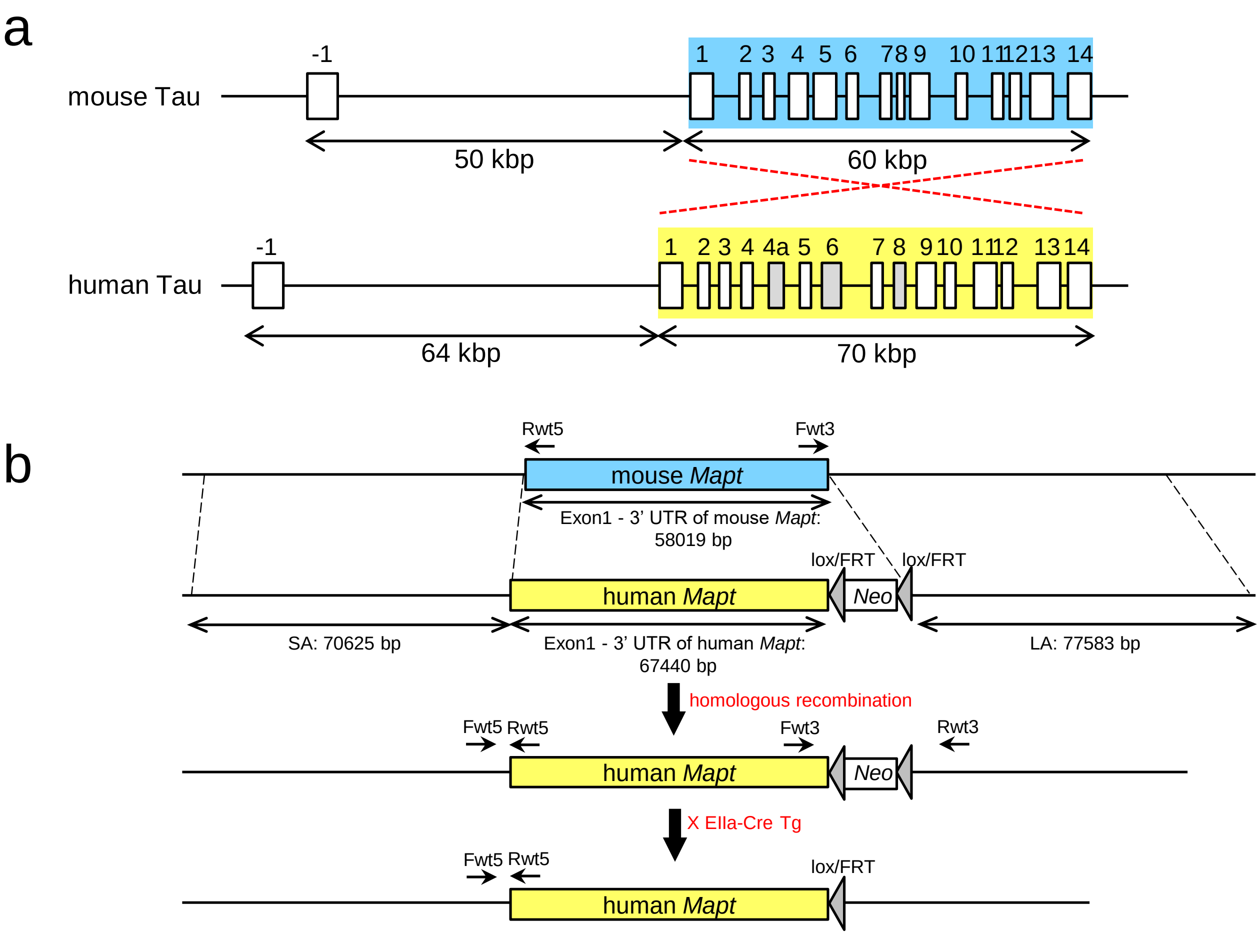

Supplementary Figure 1. Scheme of *MAPT* (hTau) KI generation
(a) The entire genomic sequence of murine *Mapt* (from exon 1 to exon 14) was substituted with the human *Mapt* sequence (from exon 1 to exon 14) by homologous recombination. (b) The human *Mapt* knock-in targeting construct is illustrated. The targeting construct contained a short arm (SA) corresponding to the 5′ mouse *Mapt* genomic flanking region, the human *Mapt* sequence (exon1-14), the neomycin-resistant gene (*neo*) flanked by *loxP* sites, and a long arm (LA) corresponding to the 3′ mouse *Mapt* genomic flanking region. The targeting construct was linearized and injected into ES-R1 cells by electroporation. After selection by G418, homologous recombination was analyzed by PCR using the following primers: Fwt5: 5'-GTCAGATCACTAGACTCAGC-3', Rwt5: 5'-CTGTGCTCCACTGTGACTGG-3', Rhm5: 5'-CTGCTTGAGTTATCTTGGCC-3', Fwt3: 5'-ATATCTCCTTCC TGACGTGG-3', Rwt3: 5'-GGGAGTCTCCTAAGATGTCC-3' and Fhm3: 5'-TGCGAGGCCAGAGGCCACTTGTGTAGC-3'. Positive clones were injected into C57BL/6 blastocysts, and the chimeric mice were crossbred with EIIa CRE transgenic mice to eliminate the *neo* gene. Before use in experiments or cross-breeding, the mice were backcrossed onto C57BL/6 mice for 5 generations.

Supplementary Figure 2

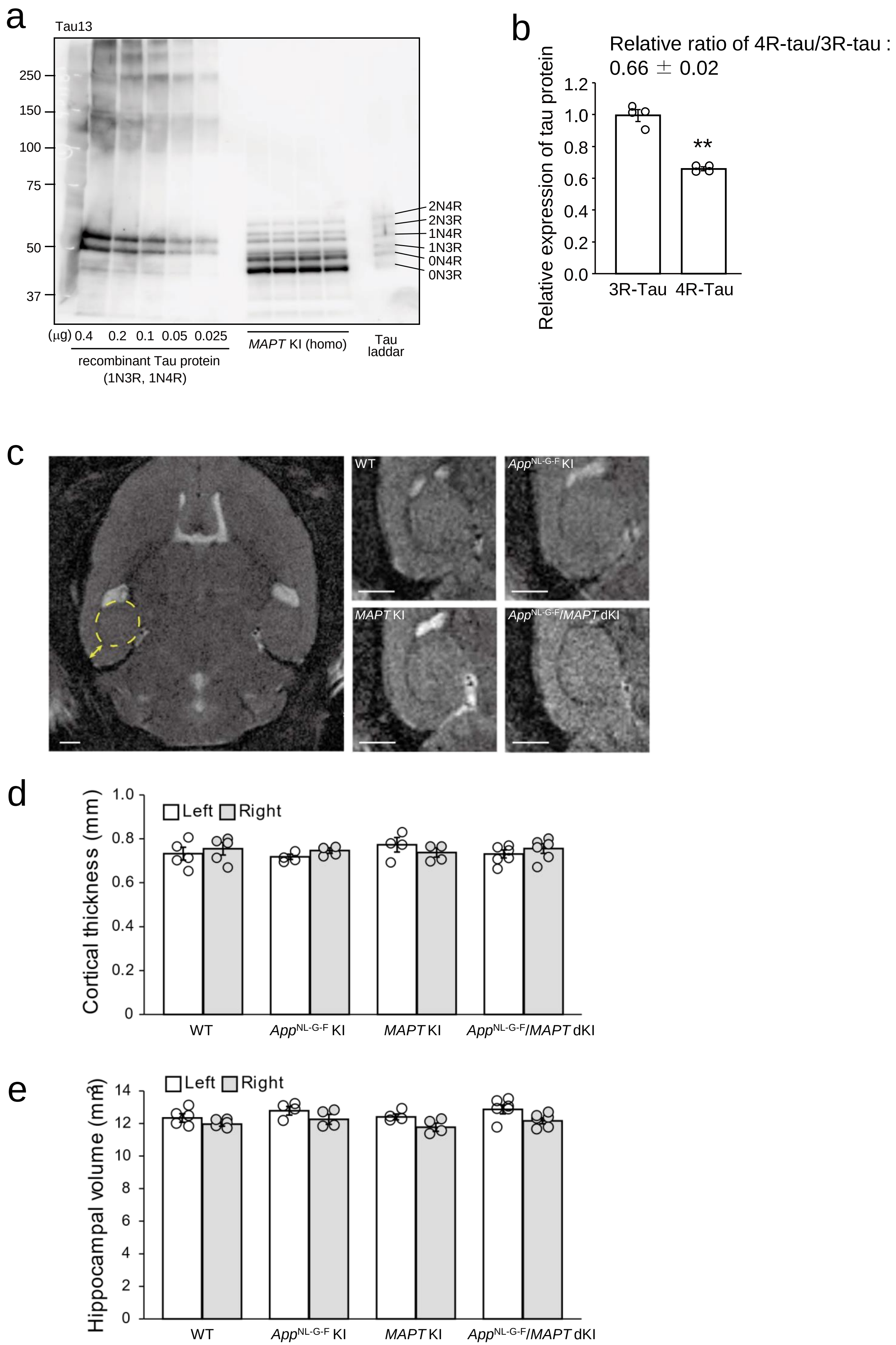

Supplementary Figure 2. Characterization of *MAPT* KI mouse

(a, b) The relative ratio of protein levels of 4R-tau/3R-tau in *MAPT* KI mice. Data represent mean ± SEM (n = 4, Student t test (t(6) = 10.098, **P = 2.74 x 10-5). (c-e) MRI-based volumetry of $App^{NL\text{-}G\text{-}F}$/*MAPT* double-KI (dKI) mouse brain. (**c**) Horizontal images of T2-weighted 1H MRI scan of 21-month-old mouse brain. The region delimited by the dotted line in the left image corresponds to the hippocampus. The right images show the zone around the left entorhinal cortex and hippocampus of corresponding mice. Scale bars represent 1 mm. (**d,e**) Thickness of entorhinal cortex (arrow in A) and hippocampal volumes of corresponding mouse strains were measured using ImageJ software. Data represent mean ± SEM (n = 5 (WT), n = 4 ($App^{NL\text{-}G\text{-}F}$ KI), n = 4 (*MAPT* KI) and n = 6 ($App^{NL\text{-}G\text{-}F}$/*MAPT* dKI); two-way ANOVA with post-hoc Scheffe's F test (left of entorhinal cortex thickness: $F(1,18) = 0.190$, $P = 0.188$; right of entorhinal cortex thickness: $F(1,18) = 0.060$, $P = 0.810$; left of hippocampal volume: $F(1,18) = 3.993$, $P = 0.064$; right of hippocampal volume: $F(1,18) = 1.980$, $P = 0.180$)). Source data are provided as a Source Data file.

Supplementary Figure 3

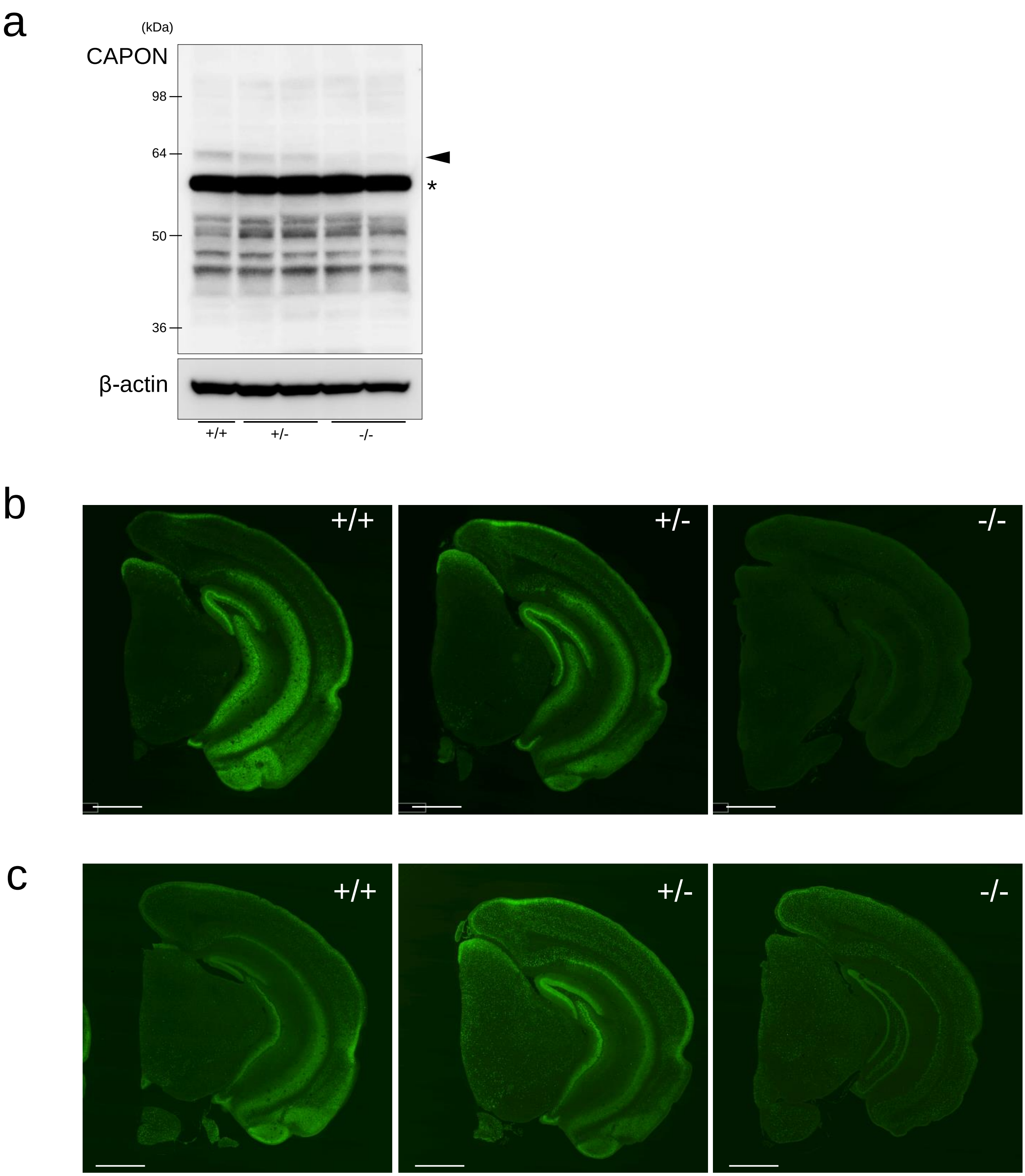

Supplementary Figure 3. Specificity of the anti-CAPON antibody for endogenous CAPON
(a) Protein samples from WT and CAPON-deficient (heterozygous and homozygous) mouse brain were separated by SDS-PAGE and transferred to membrane. CAPON signals were detected using a mouse anti-CAPON antibody (Clone#: C-9; Santa Cruz). The bands at 64kDa (arrowheads) are CAPON, whereas the lower bands (asterisks) are nonspecific. (b) Paraffin sections of WT and CAPON-deficient (heterozygous and homozygous) mouse brain were immunostained by anti-CAPON antibody (Clone#: C-9). The antibody specifically detected endogenous CAPON in WT and heterozygous mice, and was used for immunohistochemistry, except in Fig.7a. Scale bar: 1cm (c) Paraffin sections were immunostained using anti-CAPON antibody produced in rabbit (Clone#: R300; Santa Cruz). This antibody was used for Duolink, as illustrated in Fig.7a. Scale bar: 1cm.

Supplementary Figure 4

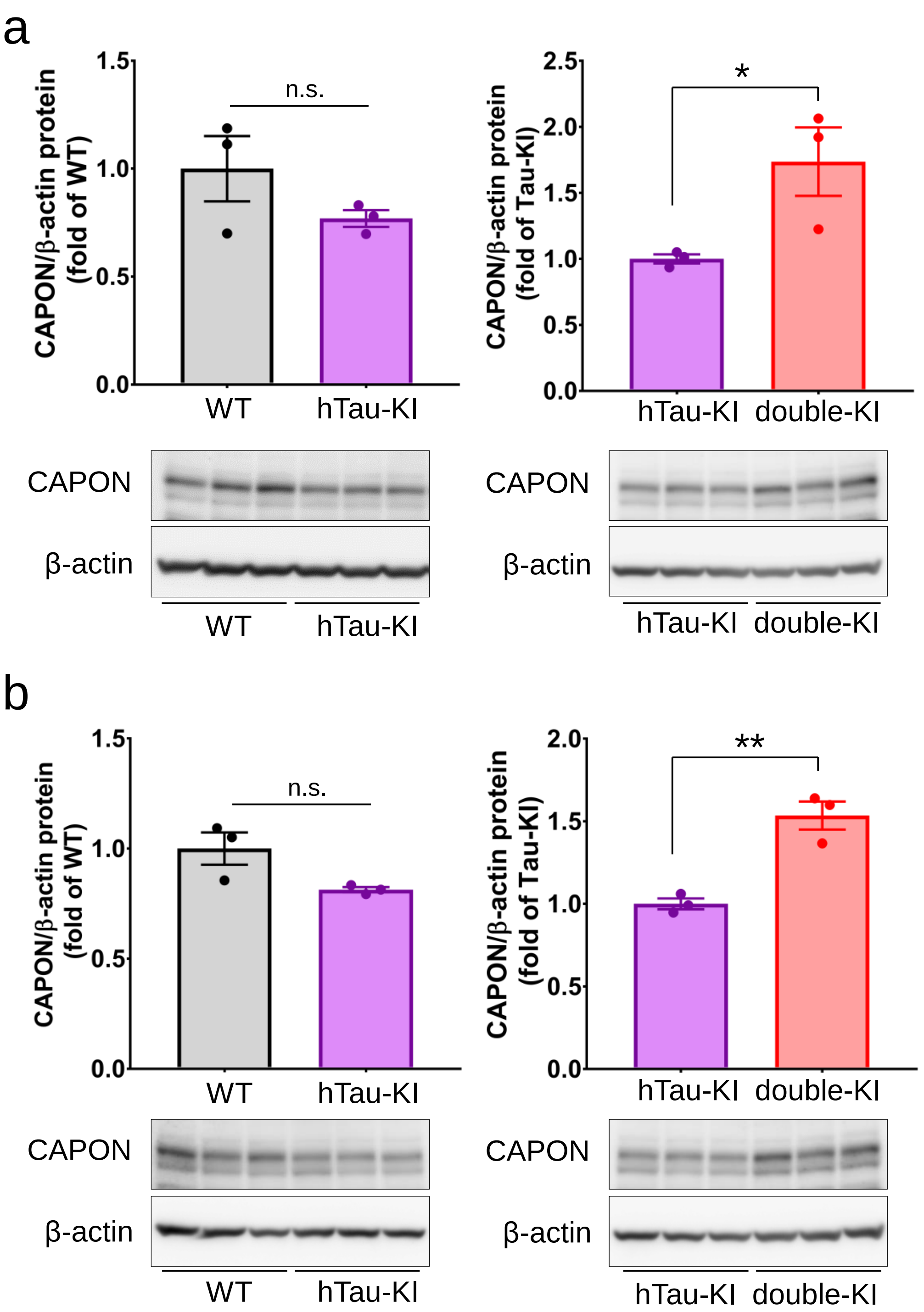

Supplementary Figure 4. Expression level of CAPON in hTau-KI and *App*$^{NL\text{-}G\text{-}F}$ X hTau-double-KI
(a,b) The protein levels of CAPON in the hippocampi (a) and cortices (b) of 12-month-old WT vs hTau-KI and hTau-KI vs double-KI male mice were compared. Values shown in the graph represent the band intensity of CAPON divided by the intensity of β-actin, expressed as the mean level ± SEM (n=3, *p<0.05, **p<0.01). Source data are provided as a Source Data file.

Supplementary Figure 5

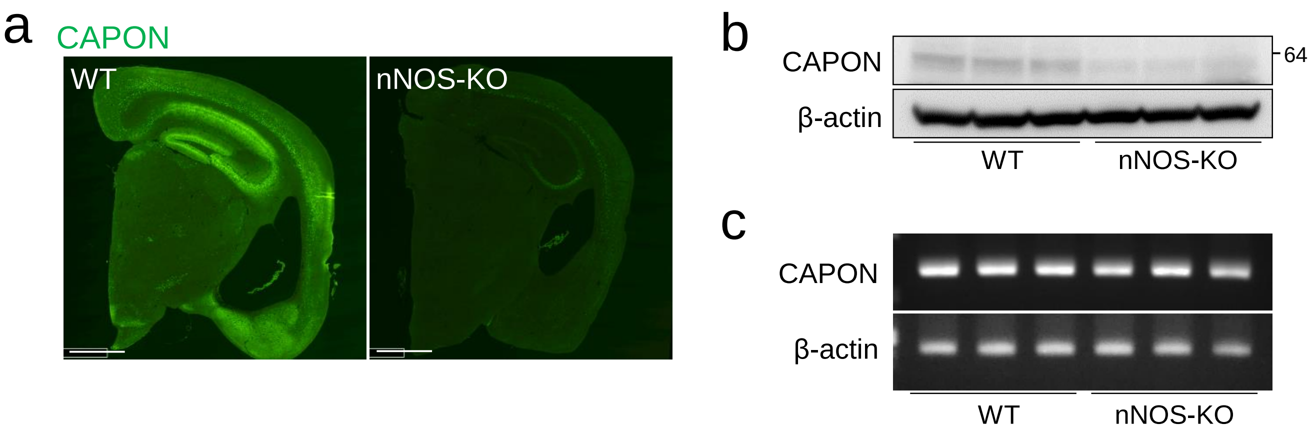

Supplementary Figure 5. CAPON expression in nNOS-deficient mice

(a) Brain sections from WT and nNOS-knockout (KO) mice were immunostained by anti-CAPON antibody. CAPON expression was dramatically lowered in the nNOS-KO mice compared to WT. Scale bar: 1mm (b) The protein level of CAPON in the nNOS-KO mouse brain was lower than that in the WT by western blot. (c) There was no difference in the mRNA levels of CAPON in the WT and nNOS-KO mouse brain, as determined by semi-quantified RT-PCR. These results suggest that CAPON protein but not its mRNA is more unstable or exhaustively degraded in the nNOS-deficient mouse.

Supplementary Figure 6

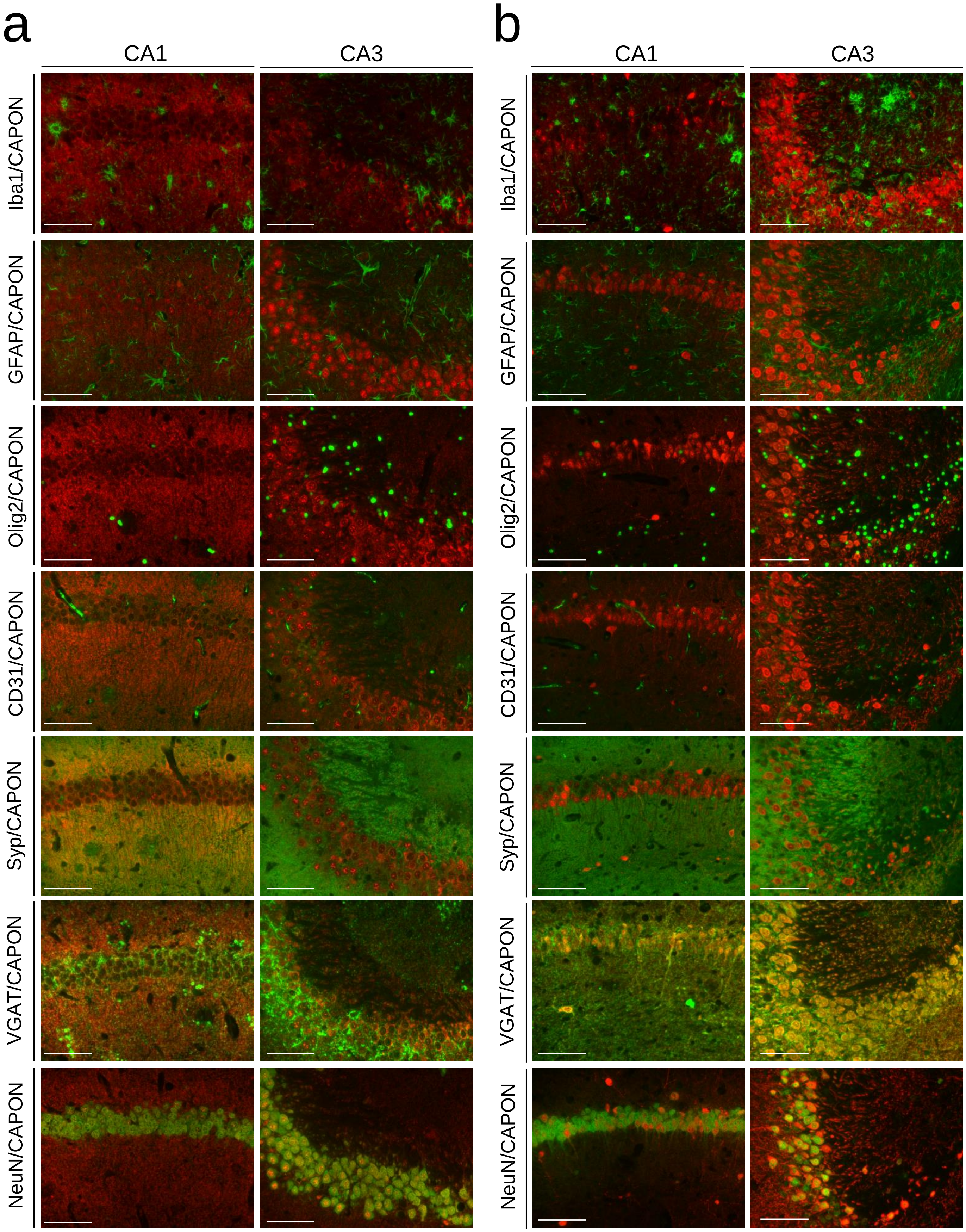

Supplementary Figure 6. Endogenous and AAV-introduced CAPON expresses in neuronal cells
(a,b) Representative image of hippocampal CA1 and CA3 region of control (a) and AAV-CAPON introduced mice (b) co-immunostained with CAPON (red) and cellular markers (green). Each marker protein is expressed in following type of cells; Iba1: microglia, GFAP: astrocyte, Olig2: Oligodentrocyte, CD31: endothelial cells, Syp: presynapse, VGAT: presynapse, NeuN: synapse. Scale bar: 100μm

Supplementary Figure 7

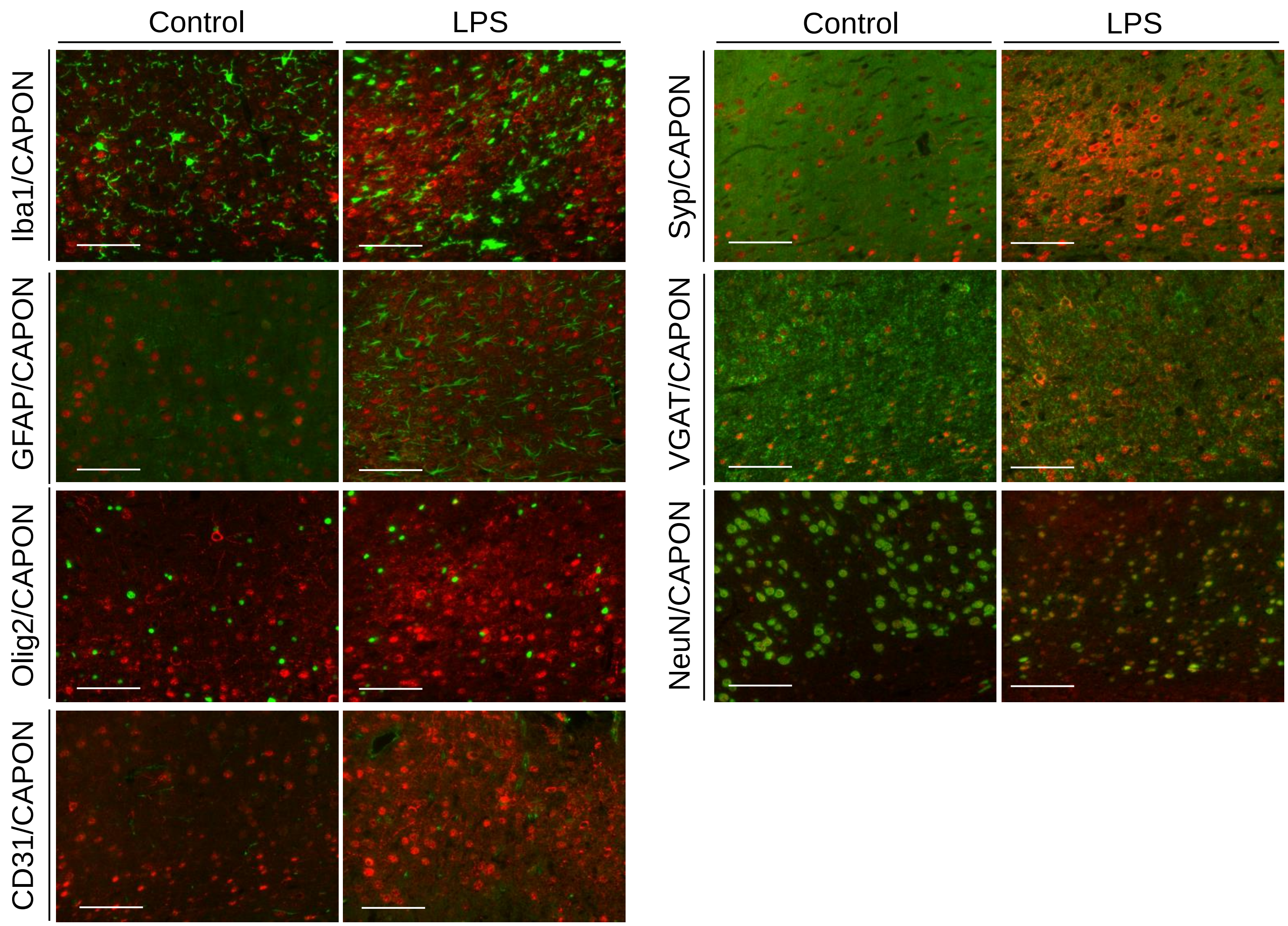

Supplementary Figure 7. LPS increase CAPON in neuronal cells but not in glial and endothelial cells
Representative image of cortical region of control and LPS introduced mice co-immunostained with CAPON (red) and cellular markers (green). Each marker protein is expressed in following type of cells; Iba1: microglia, GFAP: astrocyte, Olig2: Oligodentrocyte, CD31: endothelial cells, Syp: presynapse, VGAT: presynapse, NeuN: synapse. Scale bar: 100μm

Supplementary Figure 8

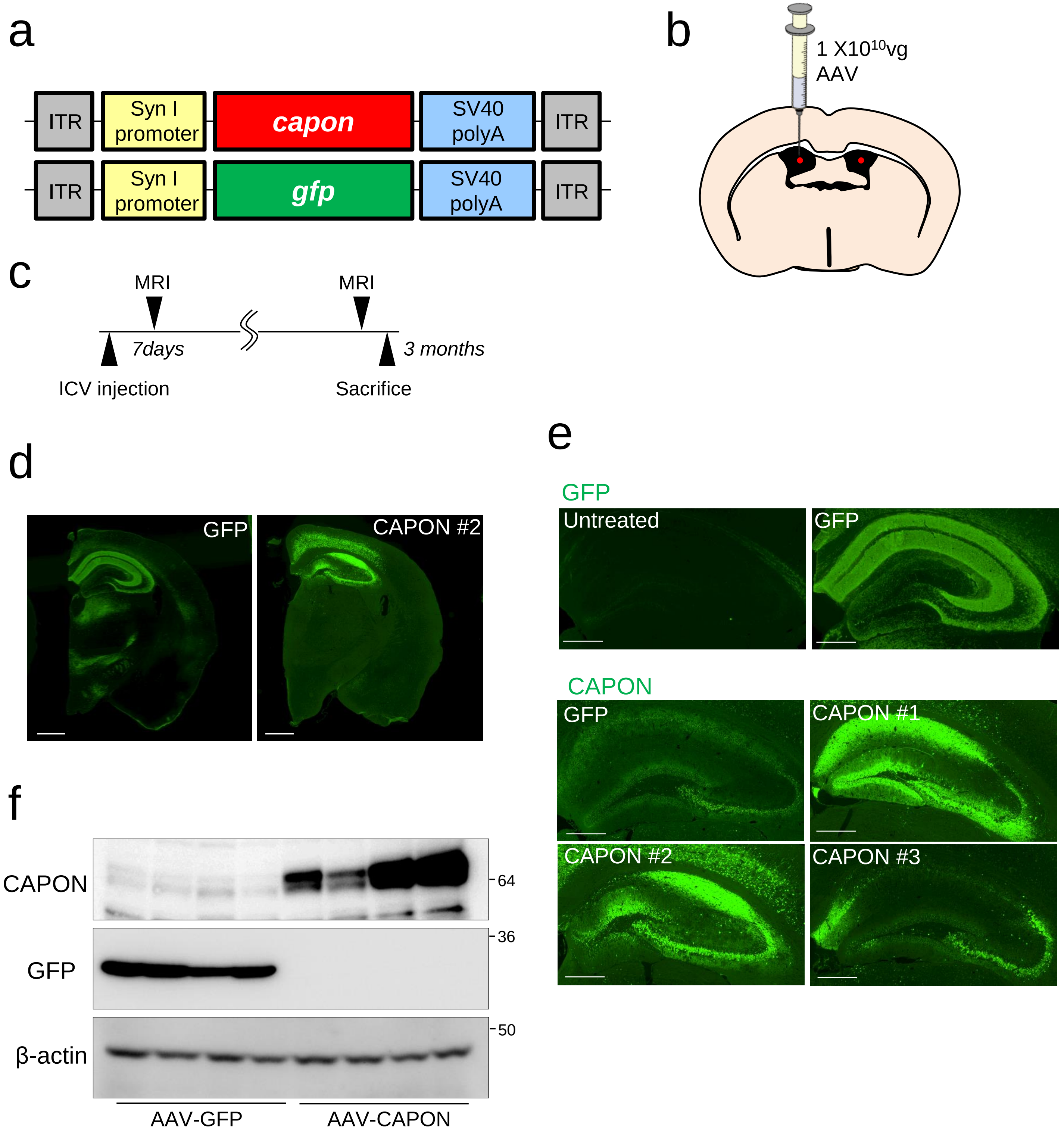

Supplementary Figure 8. Overexpression of CAPON by AAV-mediated gene introduction

(a) Structure of AAV vectors for gene overexpression. CAPON or GFP (Control) was expressed under the synapsin I (Syn I) promoter. ITR, inverted terminal repeats of AAV3. (b) An AAV solution (1×10^{10} vg/5 μL) (b) An AAV solution ($1X10^{10}$ vg/5 μL) was injected bilaterally into the ventricles (red dots). (c) In our experiments (except for Fig.7f-h, and Fig.4b-d), pathological changes were analyzed 3 months after AAV injection, and MRI was performed 7 days and 3 months after injection. The age of the mice is provided in each figure legend. (d,e) AAV-GFP or AAV-CAPON was introduced into 12-month-old male double-KI mice. Three months after AAV injection, overexpression of GFP (upper) or CAPON (lower) was detected by immunohistochemistry. The figures show the hippocampal area in untreated and AAV-GFP- or AAV-CAPON-expressing mice. Scale bar: 1mm (d) or 500µm (e). (f) Overexpression of GFP or CAPON was detected by western blotting. The band at 64k was increased by the introduction of AAV-CAPON. Source data are provided as a Source Data file.

Supplementary Figure 9

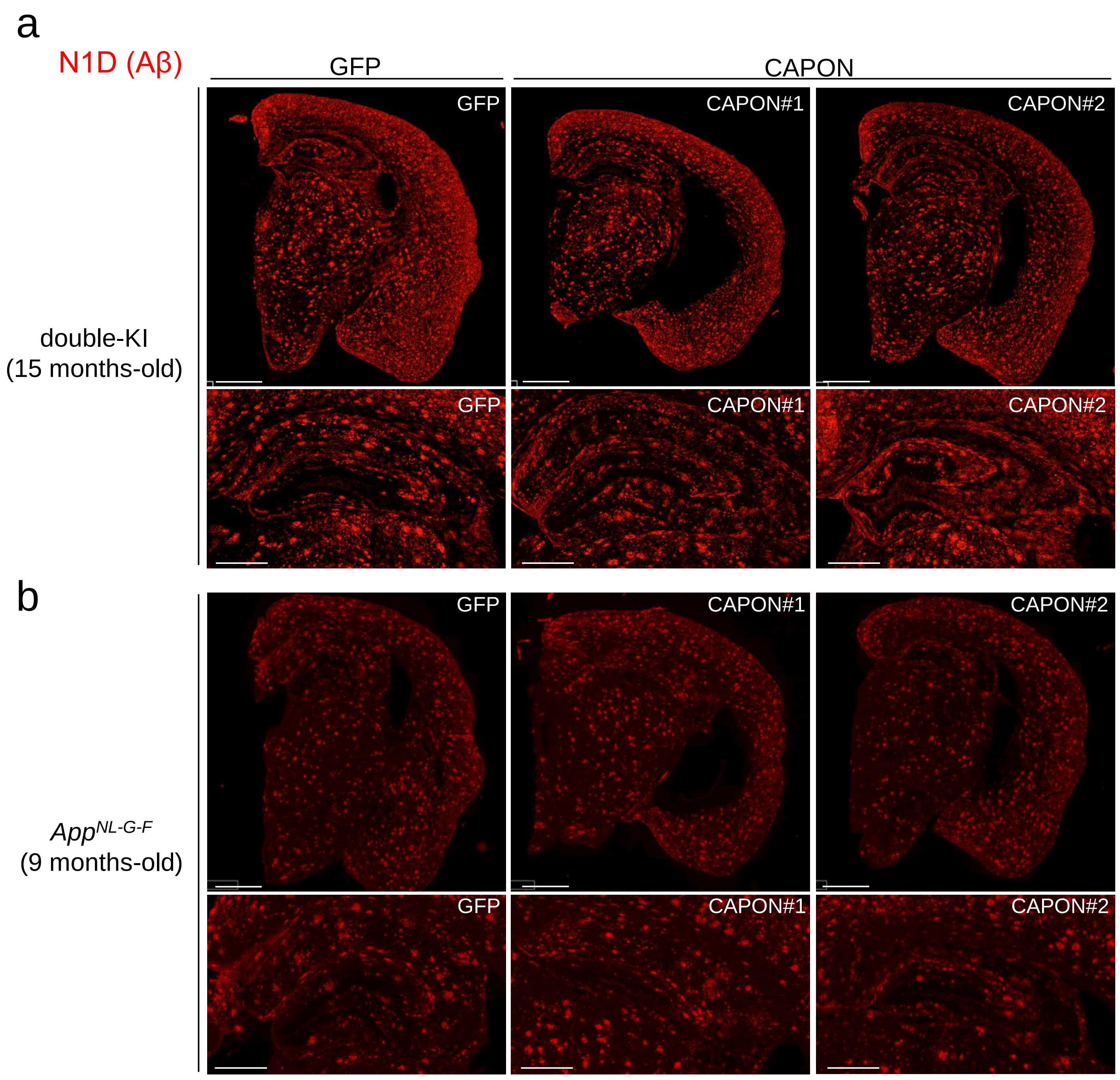

Supplementary Figure 9. Amyloid pathology in response to CAPON overexpression in double-KI and single *App*$^{NL\text{-}G\text{-}F}$-KI mice

(a, b) Brain sections from 15-month-old double-KI (a) and 9-month-old single *App*$^{NL\text{-}G\text{-}F}$-KI (b) mice overexpressing GFP or CAPON were immunostained using the N1D antibody that reacts with the N-terminus of Aβ. Each upper panel shows the whole of hemisphere, and lower panel shows hippocampal region. The amyloid pathology was not altered by CAPON overexpression in either case. Scale bar: 1mm (above) or 500μm (below) .

Supplementary Figure 10

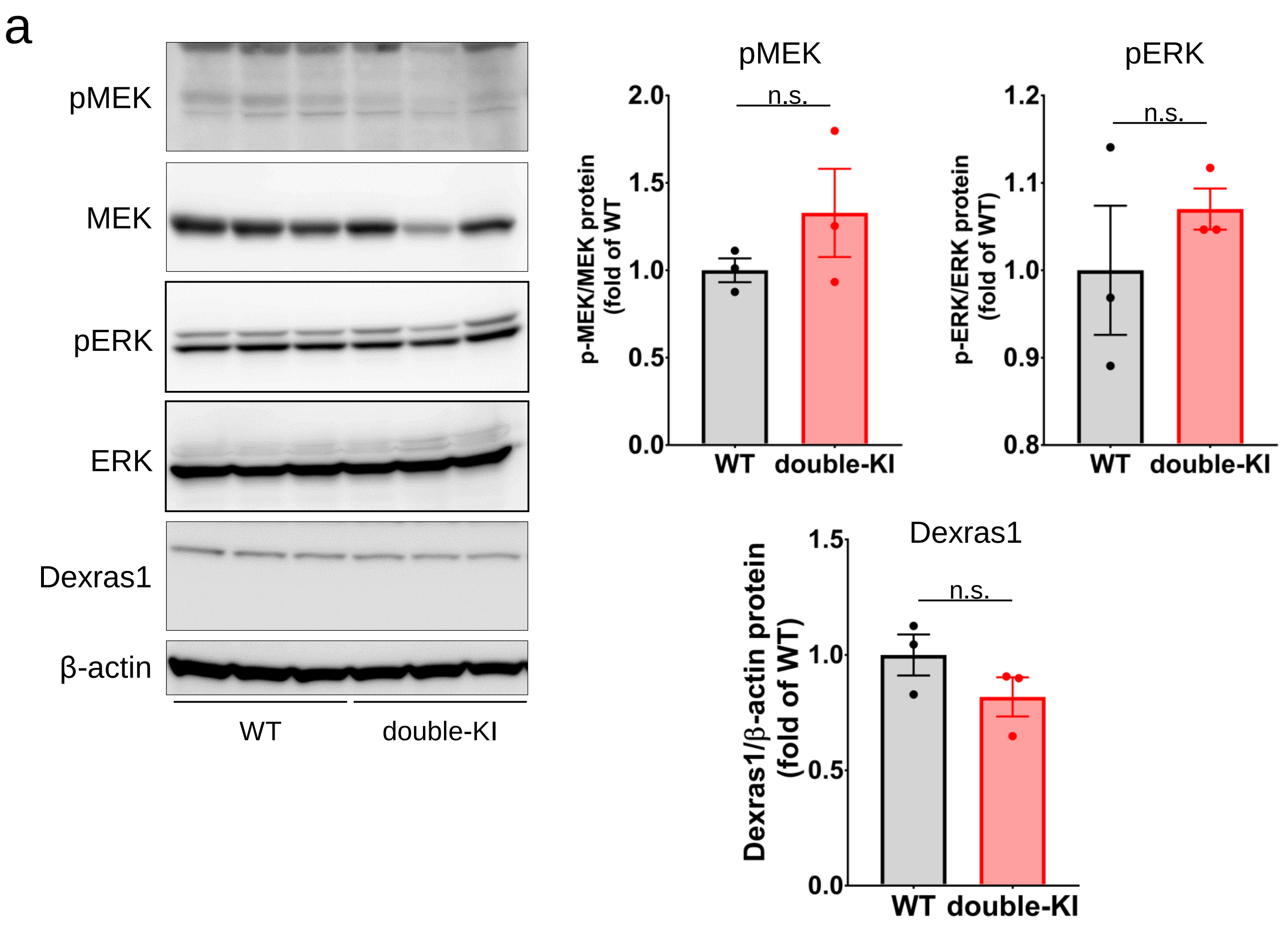

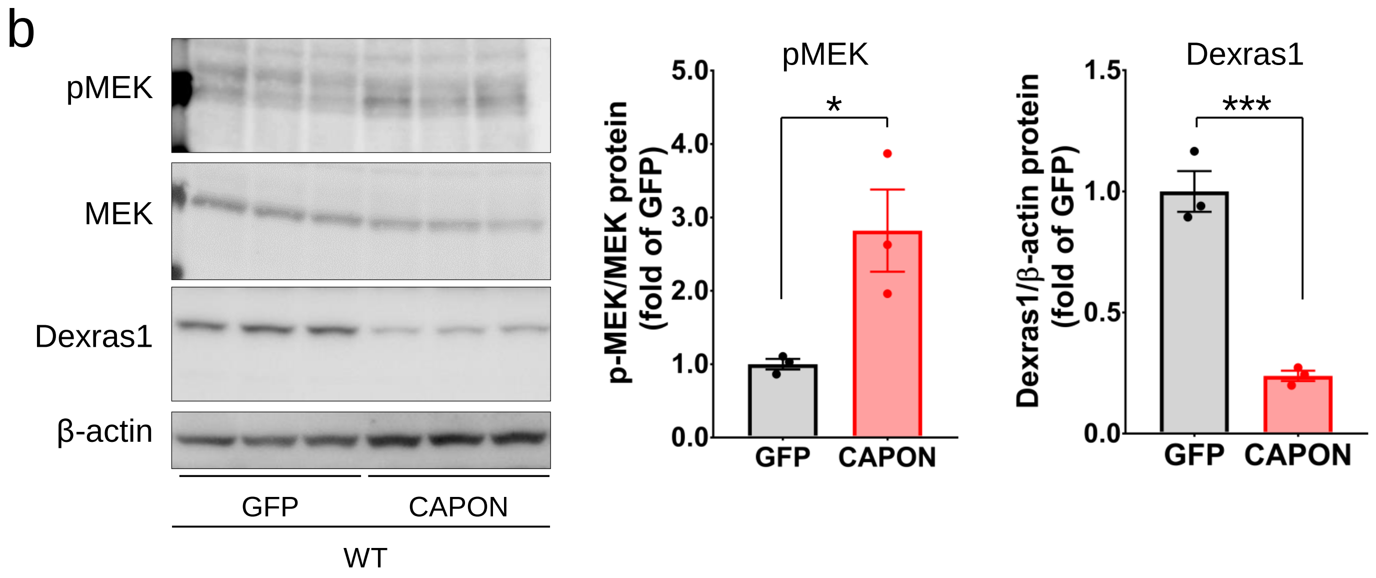

Supplementary Figure 10. CAPON-overexpression alters Dexras1 and p-MEK levels also in WT mouse

(a) The protein levels of Dexras1 and p-MEK in the hippocampi of 12-month-old WT and double-KI male mice were compared. Values show the band intensity of proteins divided by the intensity of β-actin (Dexras1), total-MEK (p-MEK) or total-ERK (p-ERK), expressed as the mean level ± SEM. (b) The protein levels of Dexras1 and p-MEK in the hippocampi of 6-month-old GFP or CAPON-overexpressed WT mice were determined (n=3, *p<0.05, ***p<0.001). Source data are provided as a Source Data file.

Supplementary Figure 11

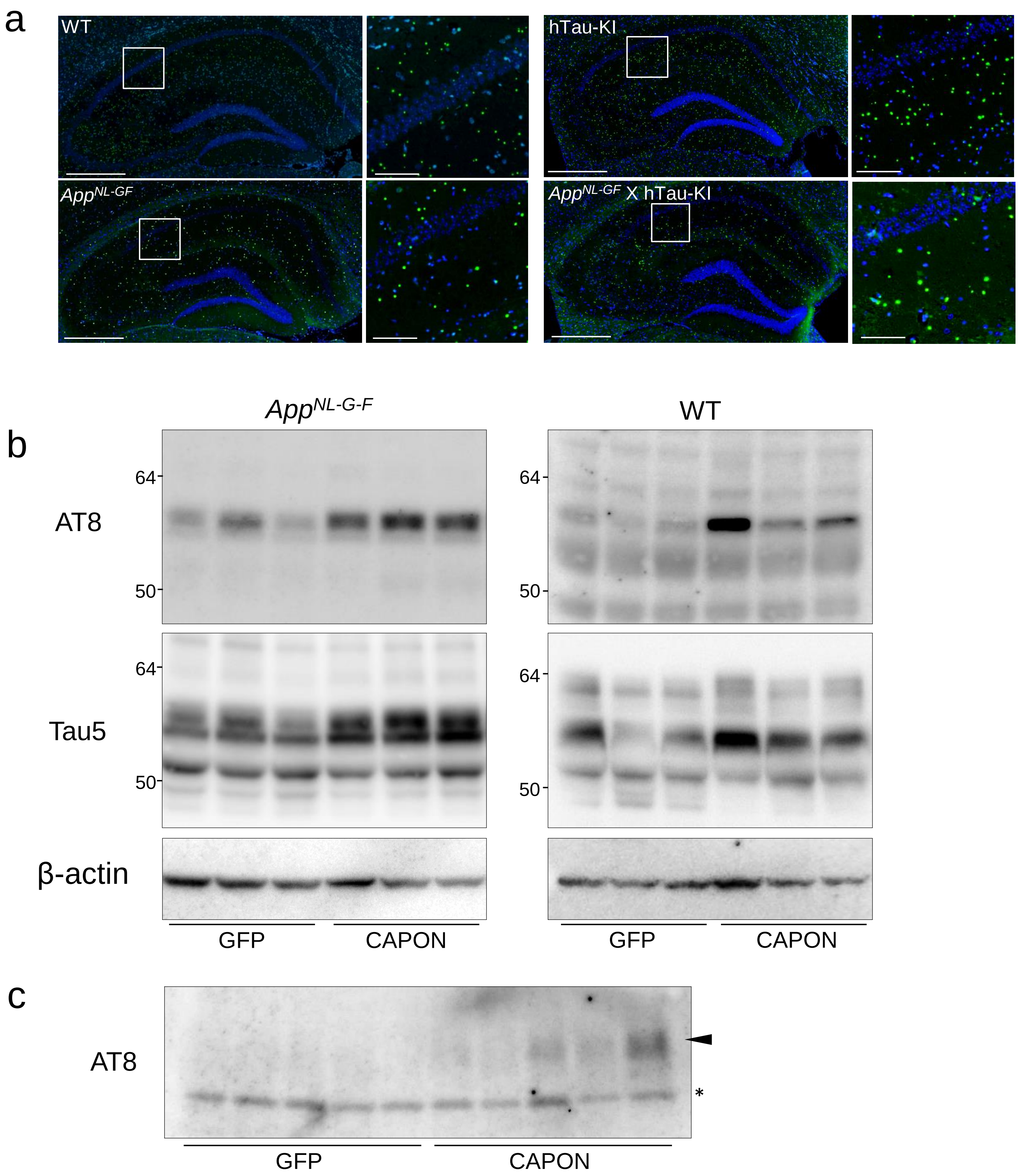

Supplementary Figure 11. CAPON overexpression in *App*$^{NL\text{-}G\text{-}F}$ single-KI or WT mice induces tau pathology (a) Duolink signals (green) generated by tau-CAPON interaction were detected in the section of 12-18-month-old WT, *App*$^{NL\text{-}G\text{-}F}$, hTau-KI, and *App*$^{NL\text{-}G\text{-}F}$/hTau double-KI male mice. Scale bar: 500μm (left) or 100μm (right) (b) The S1 fraction was prepared from the hippocampi of AAV-CAPON- or AAV-GFP-expressing *App*$^{NL\text{-}G\text{-}F}$ (left) and WT male mice (right). The level of AT8-positive tau was determined by western blot. (c) The 1% sarkosyl-insoluble fraction was prepared from AAV-CAPON- or AAV-GFP-expressing *App*$^{NL\text{-}G\text{-}F}$ mice. AT8-positive insoluble tau was detected by western blot. The arrowhead point to bands of tau. CAPON overexpression increased tau phosphorylation and insolubility. Source data are provided as a Source Data file.

Supplementary Figure 12

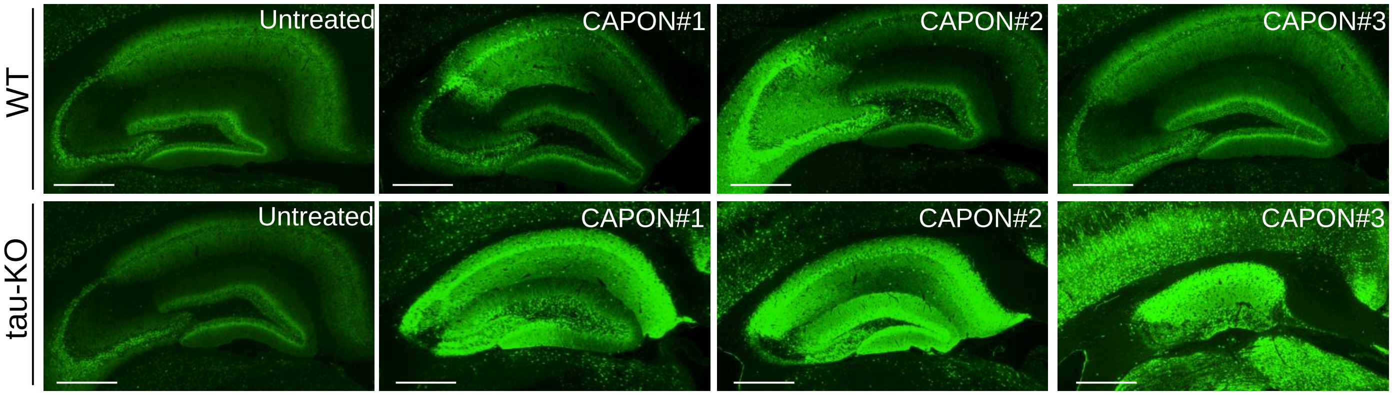

Supplementary Figure 12. CAPON overexpression in Tau-KO mice.
Brain sections from AAV-CAPON-expressing or untreated tau-KO or WT mice were immunostained by CAPON antibody. Individual numbers correspond to the numbers shown in Fig.8a. The AAV-mediated overexpression of CAPON was much higher in the tau-KO mice than in WT animals. Scale bar: 500µm

Supplementary Figure 13

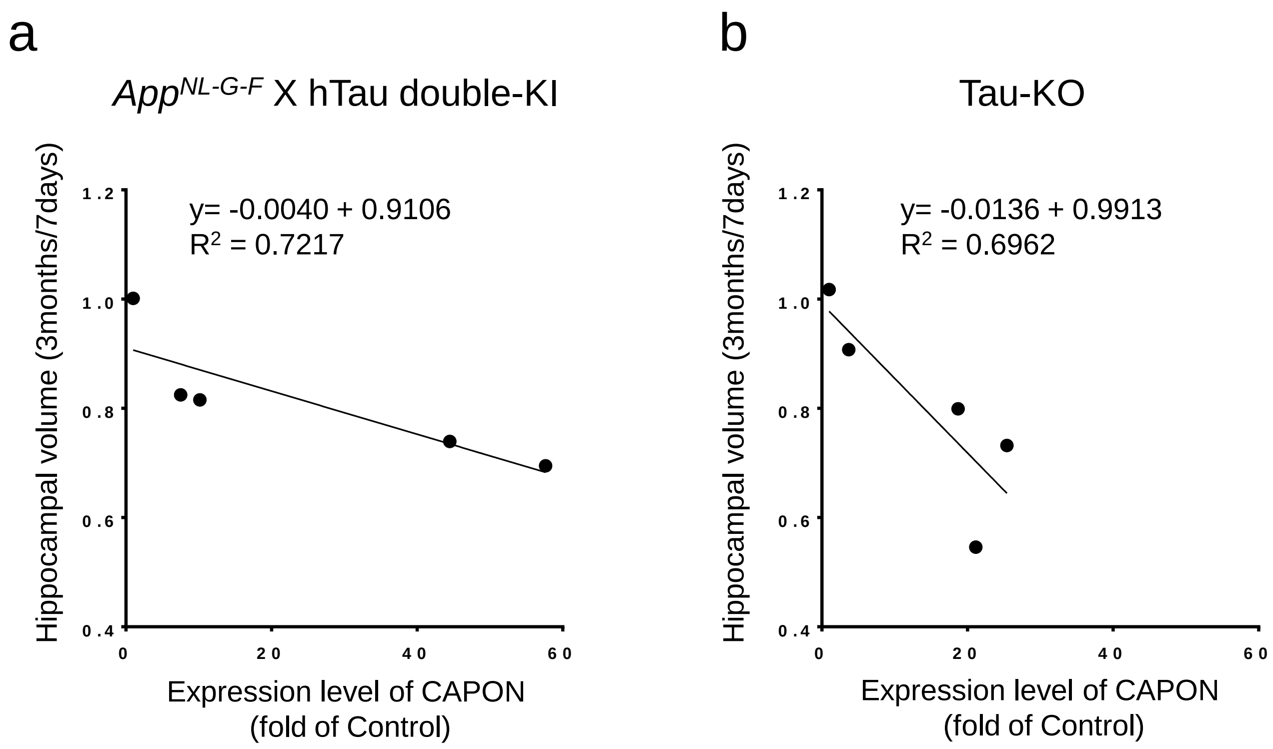

Supplementary Figure 13. Negative correlation between hippocampal volume and the overexpression level of CAPON

(a, b) Graphs show scatter plots of the reduction in hippocampal volume and the overexpression level of CAPON in AAV-CAPON-expressing double-KI (a) and Tau-KO (b) mice, respectively. The overexpression levels of CAPON were calculated from the western blots shown in Fig.3e and Fig.8a. The reduction rate was calculated as the quotient of the hippocampal volume at 3 months divided by that 7 days after AAV injection, as shown in Fig.4b and Fig.8c.

Supplementary Figure 14

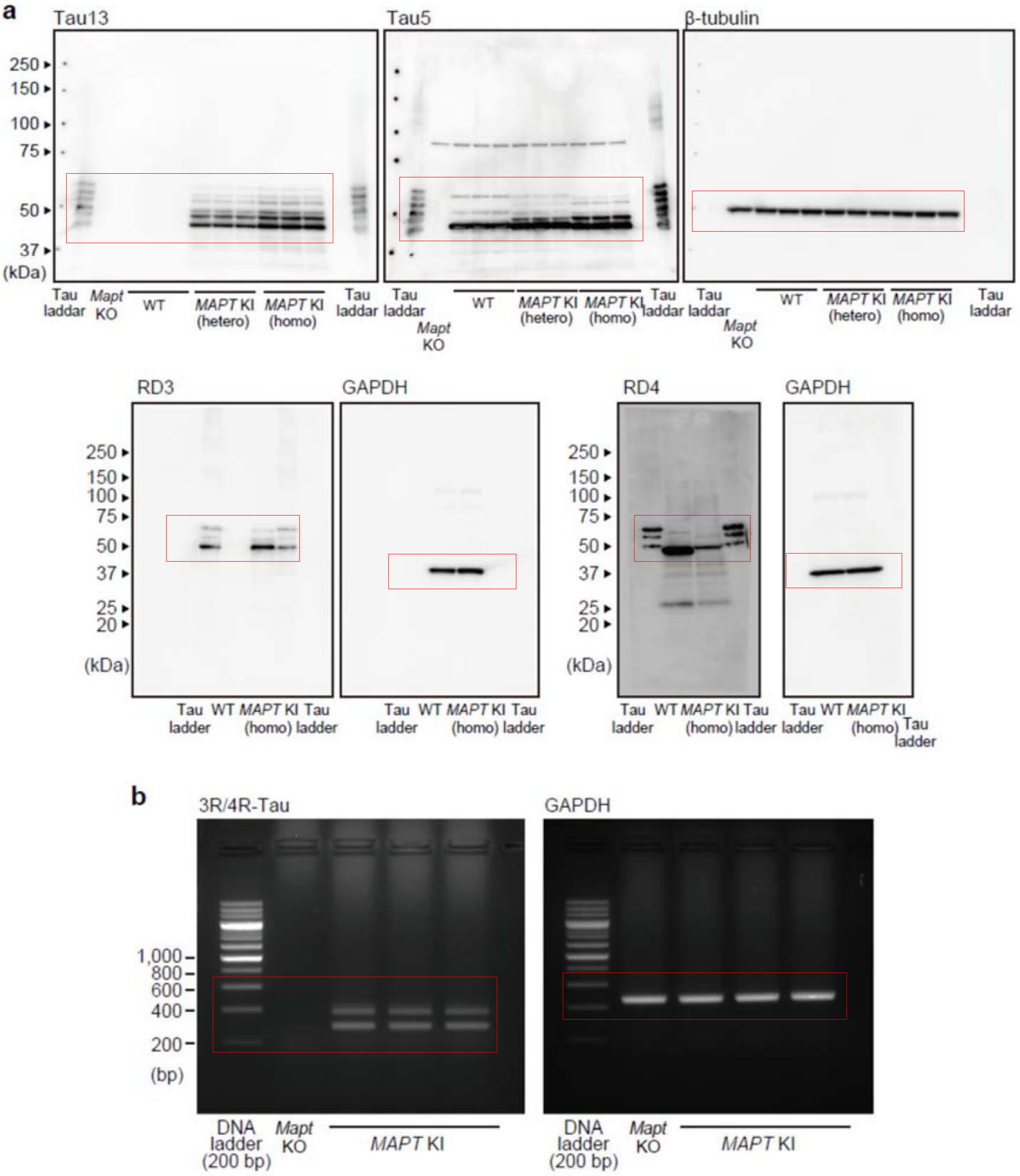

Supplementary Figure 14. The full image for Fig.1a, b
Red frames are regions shown in Fig.1a, b

Supplementary Figure 15

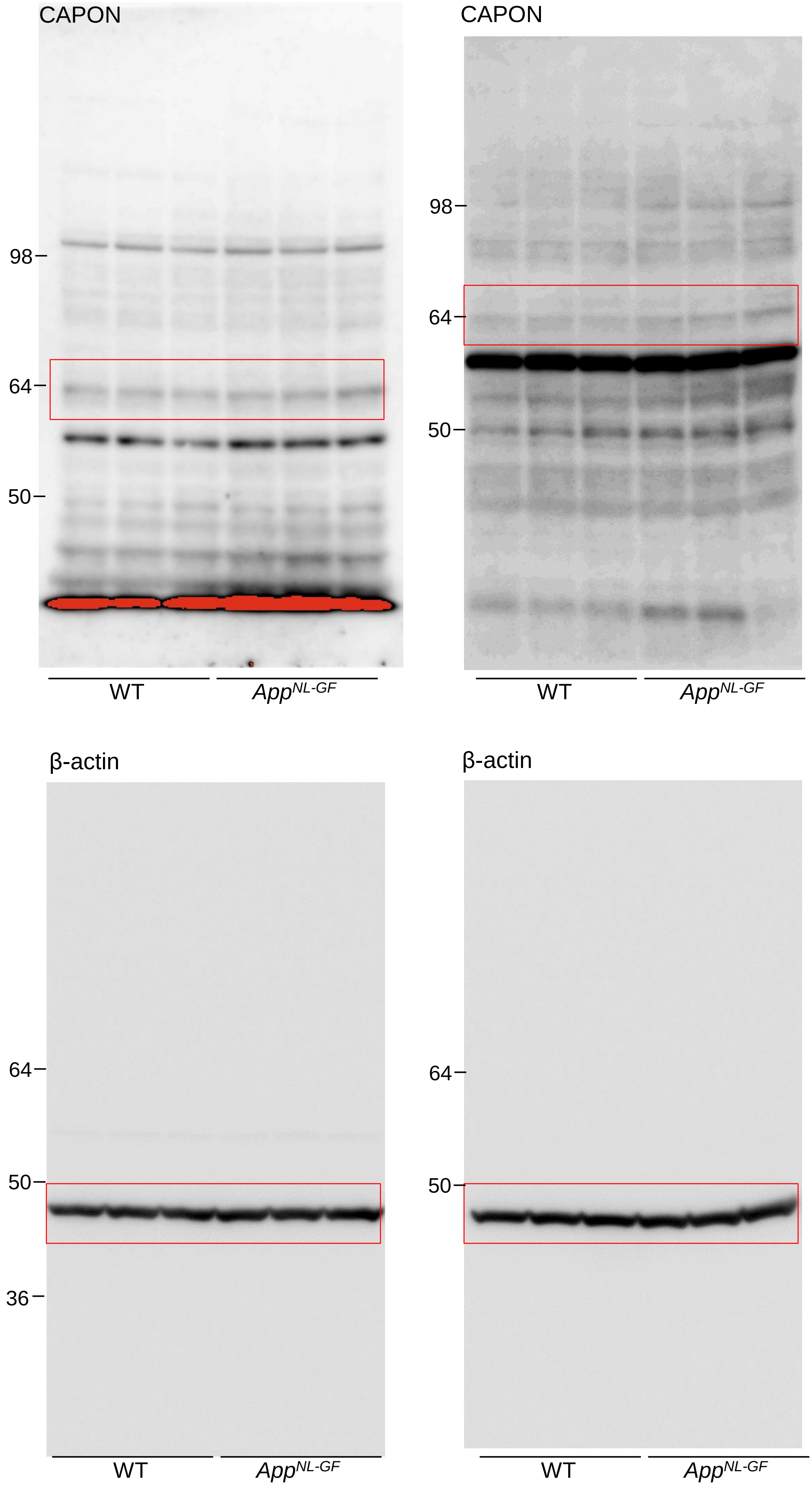

Supplementary Figure 15. The full image for Fig.2a
Red frames are regions shown in Fig.2a.

Supplementary Figure 16

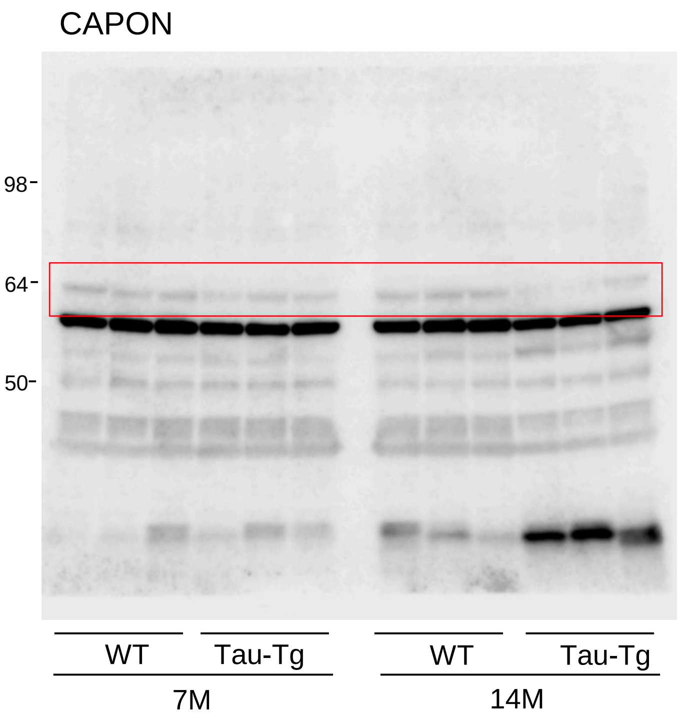

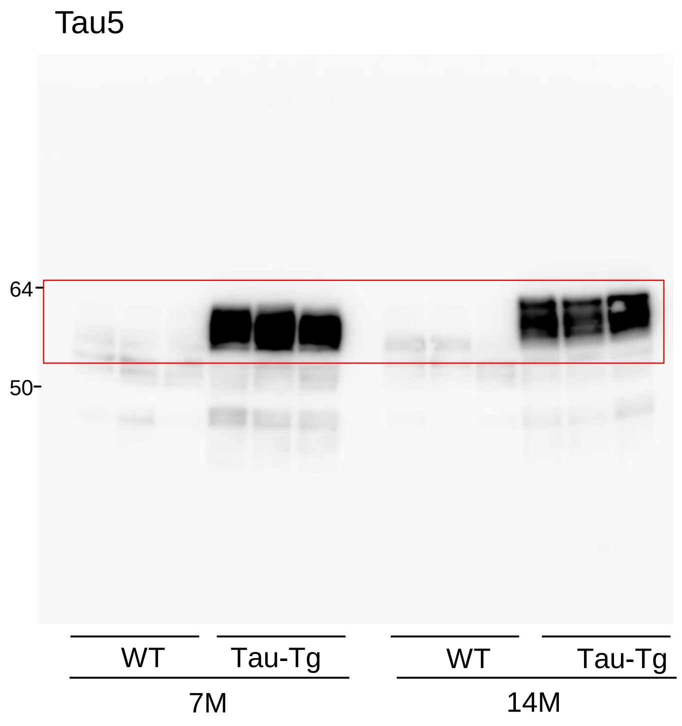

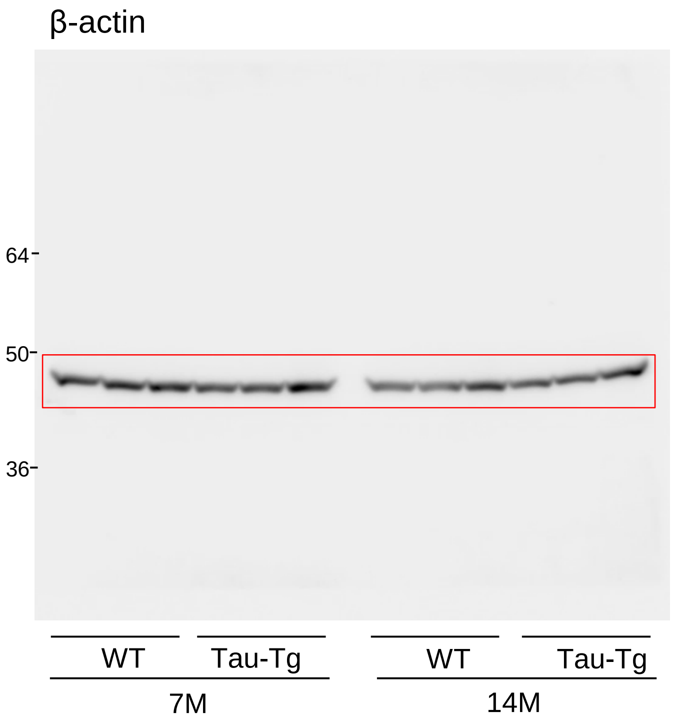

Supplementary Figure 16. The full image for Fig.2c
Red frames are regions shown in Fig.2c

Supplementary Figure 17

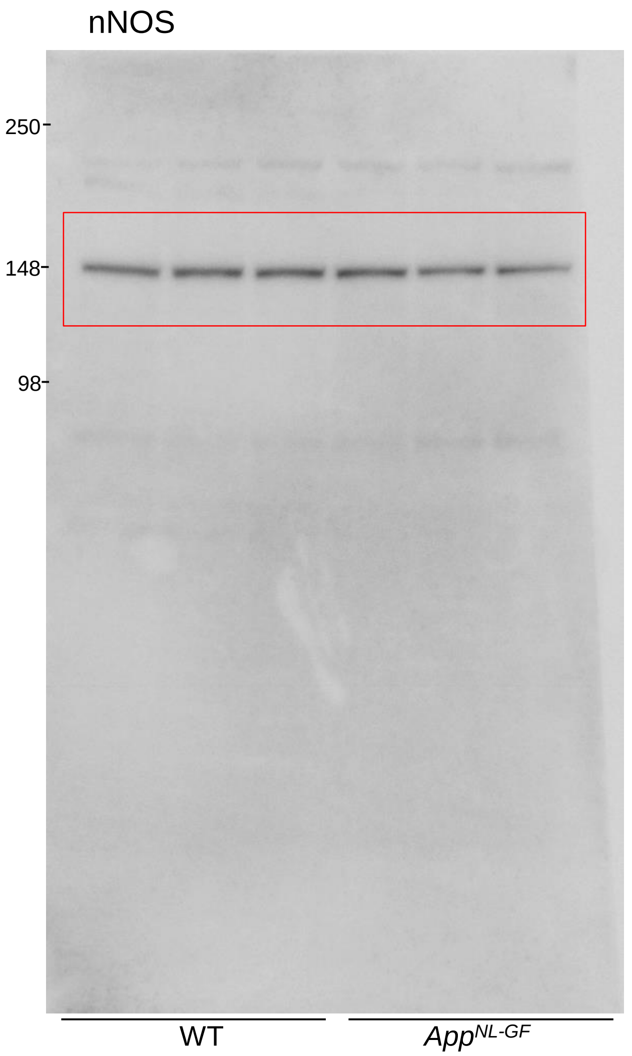

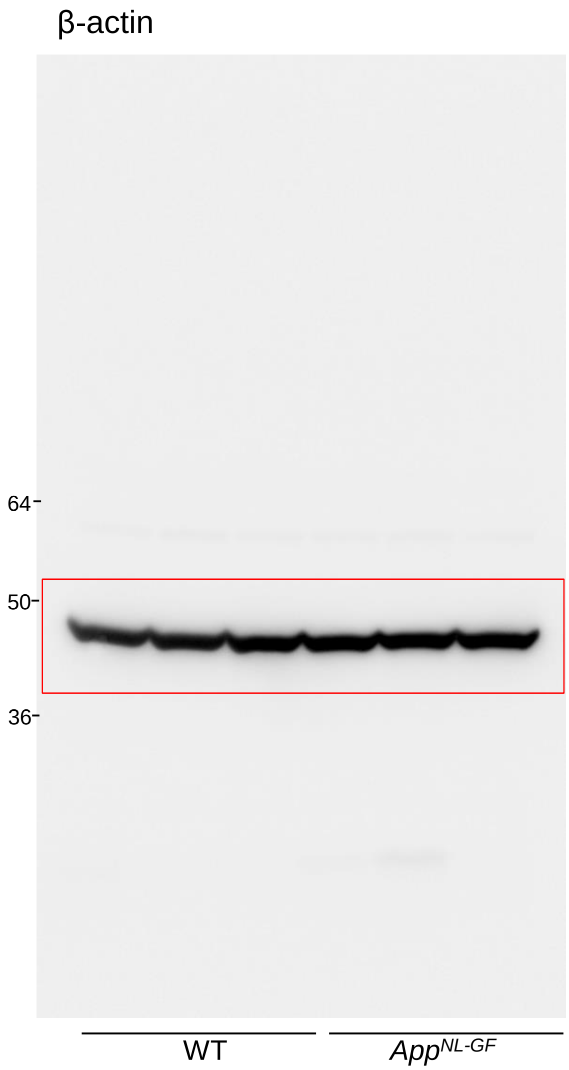

Supplementary Figure 17. The full image for Fig.3b
Red frames are regions shown in Fig.3b

Supplementary Figure 18

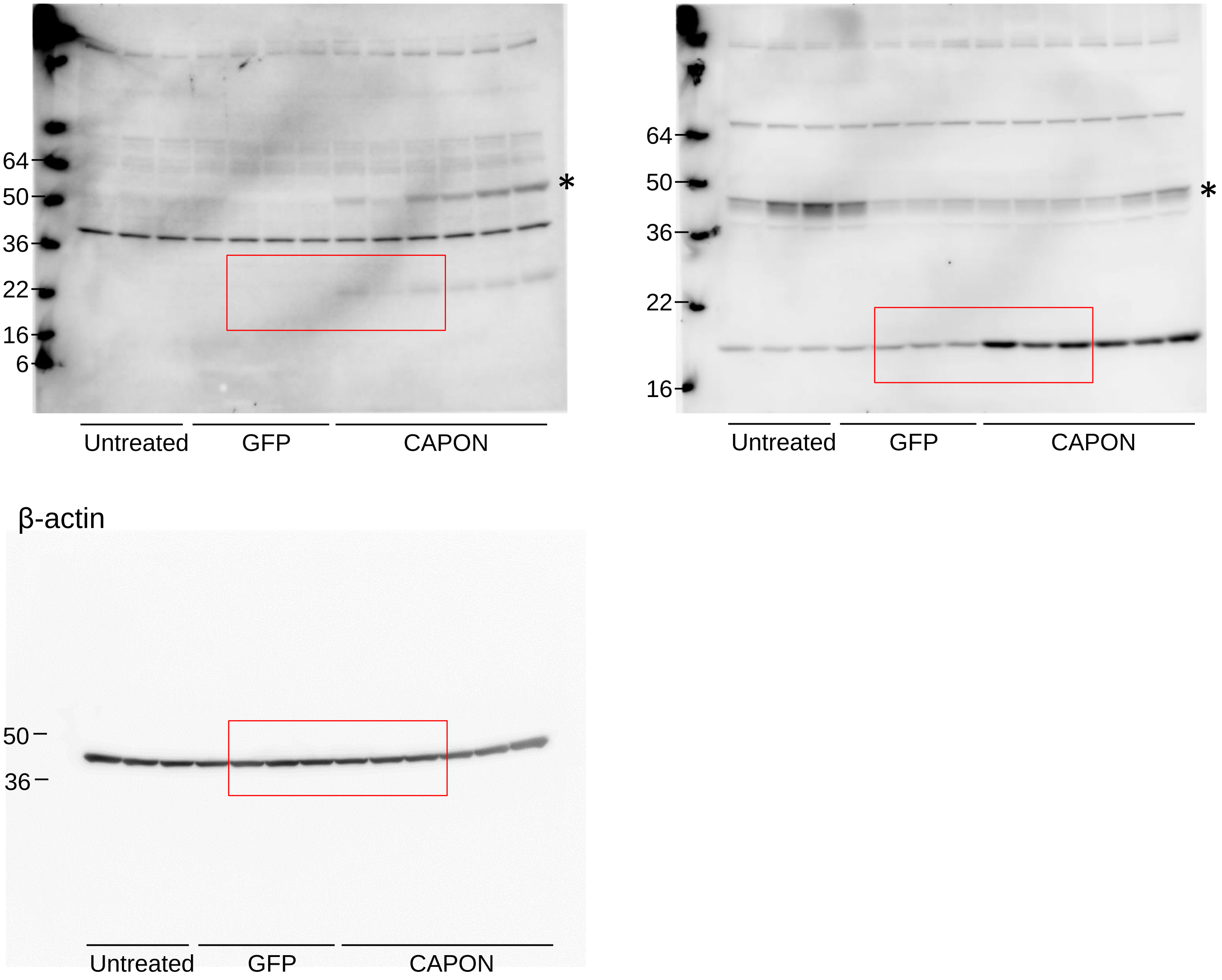

Supplementary Figure 18. The full image for Fig.4g
Red frames are regions shown in Fig.4g. Asterisks show precursor of GSDMD and GSDME, respectively

Supplementary Figure 19

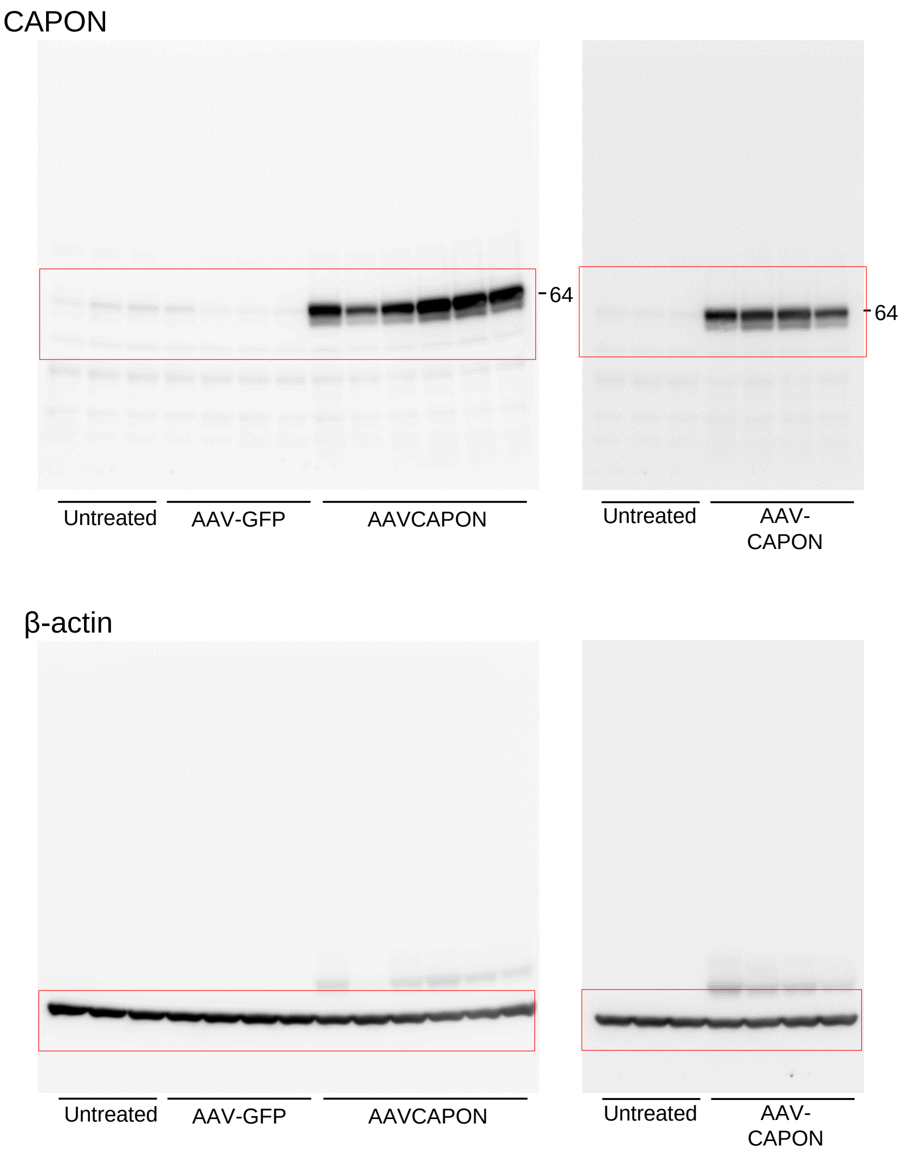

Supplementary Figure 19. The full image for Fig.5b
Red frames are regions shown in Fig.5b.

Supplementary Figure 20

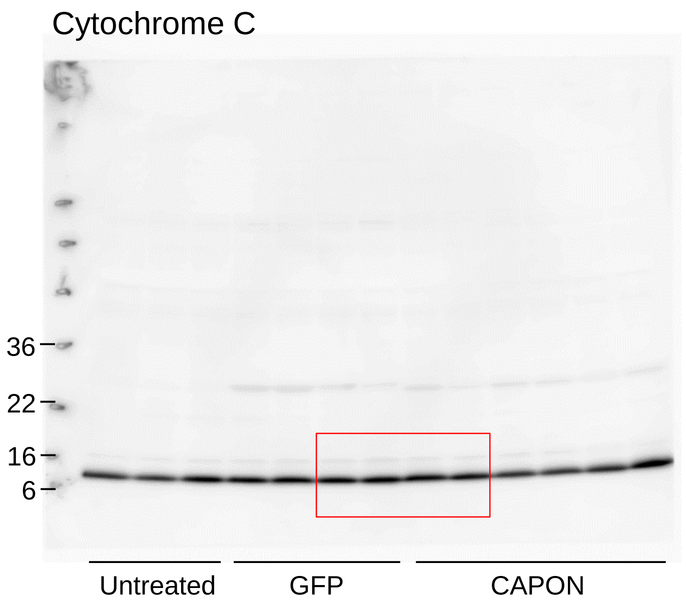

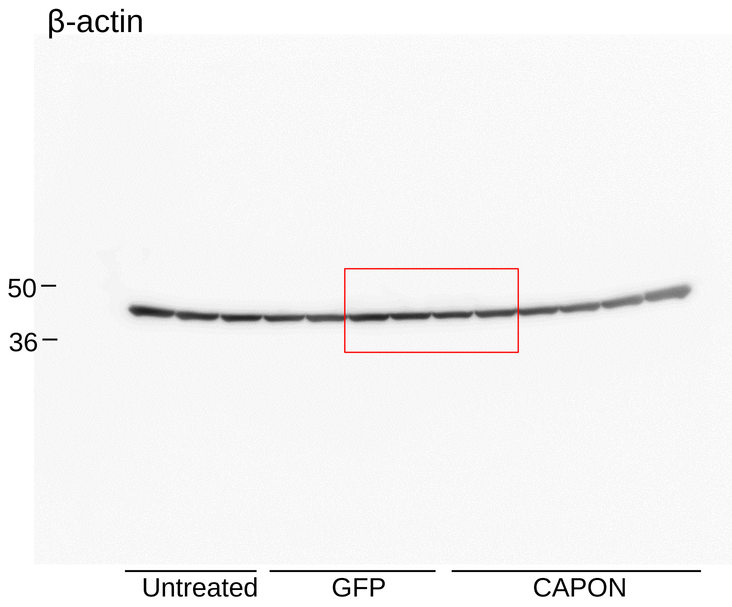

Supplementary Figure 20. The full image for Fig.6b
Red frames are regions shown in Fig.6b

Supplementary Figure 21

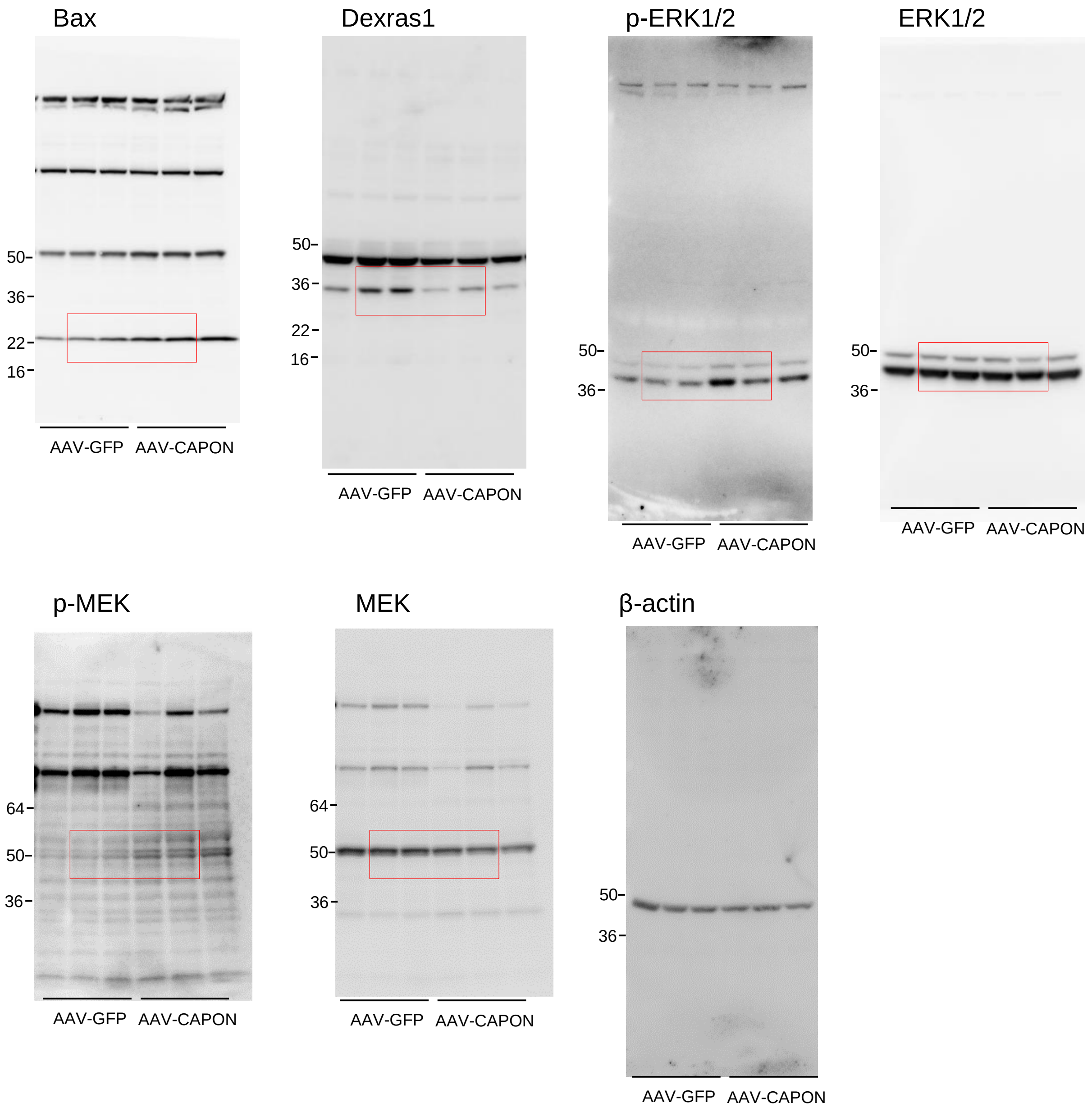

Supplementary Figure 21. The full image for Fig.6e
Red frames are regions shown in Fig.6e

Supplementary Figure 22

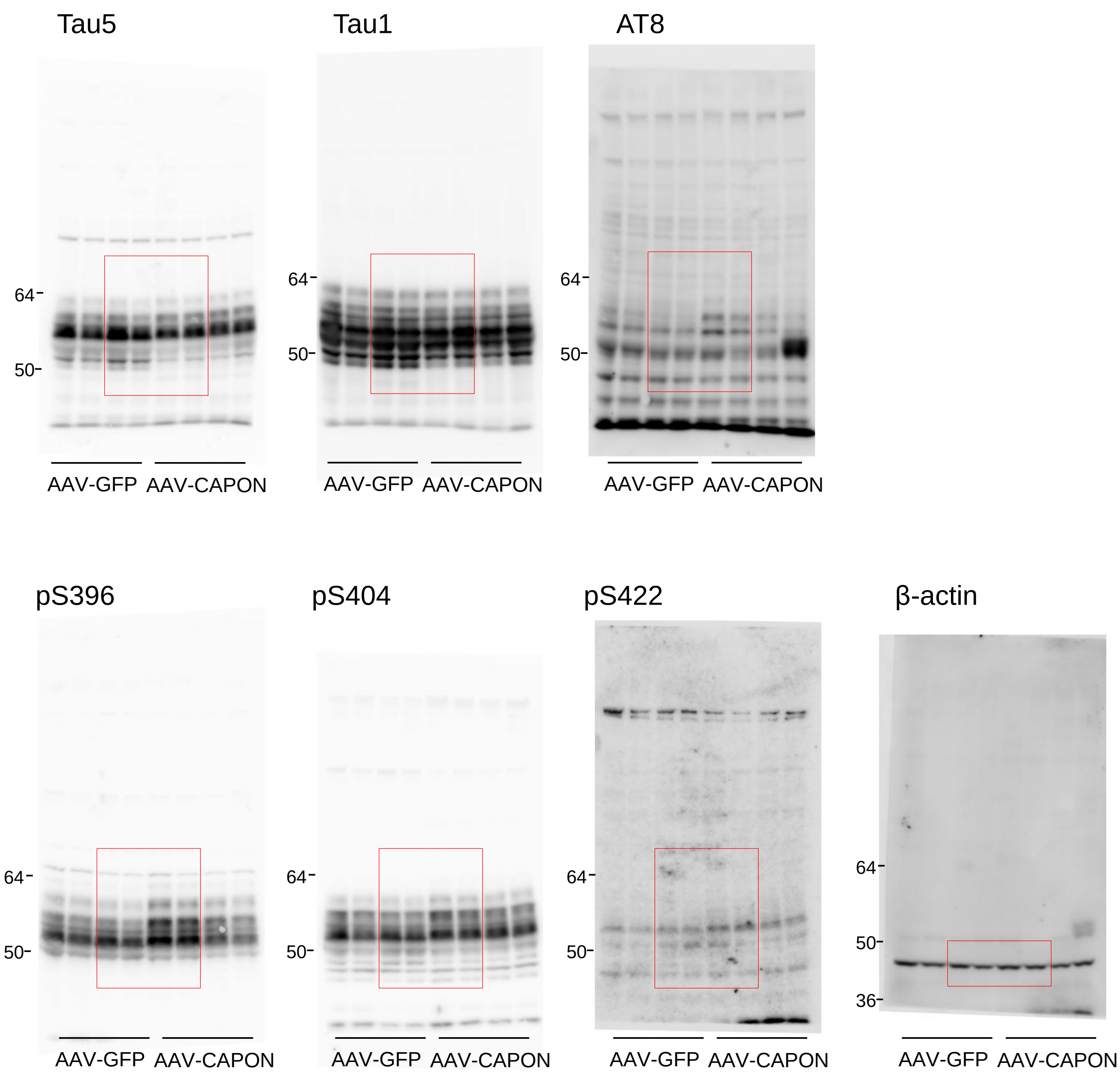

Supplementary Figure 22. The full image for Fig.7b
Red frames are regions shown in Fig.7b

Supplementary Figure 23

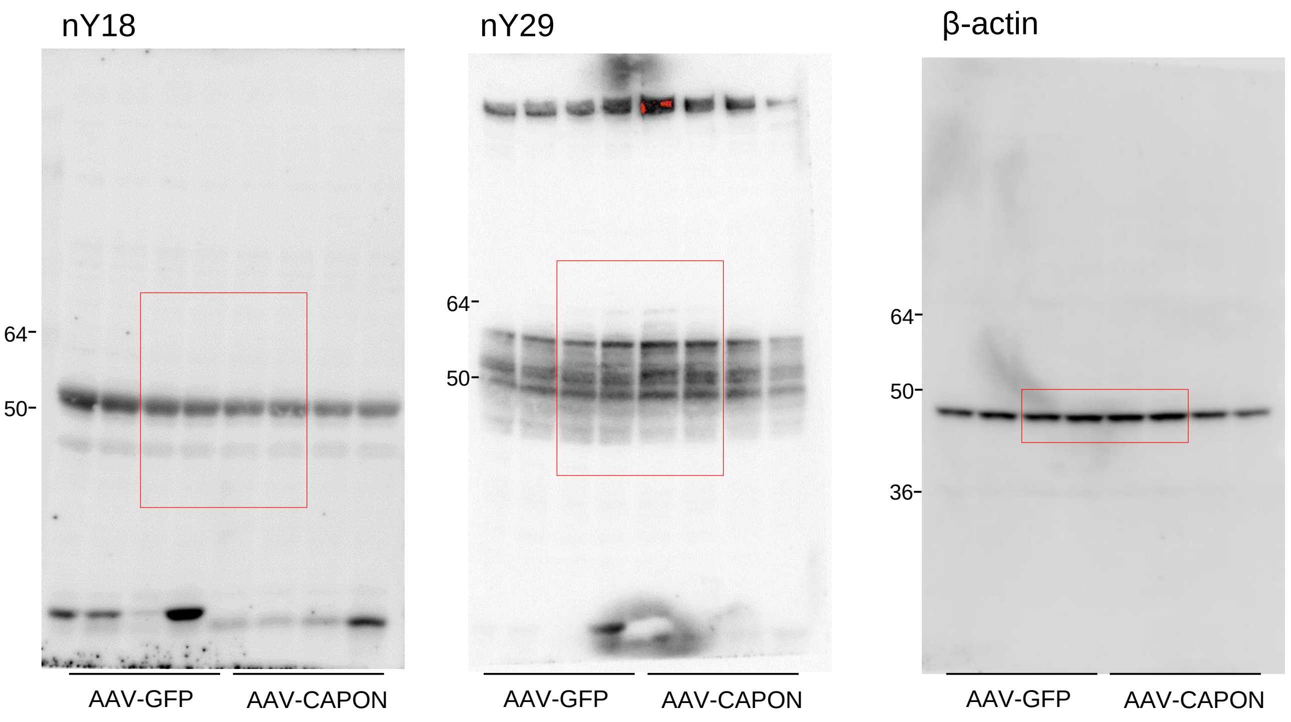

Supplementary Figure 23. The full image for Fig.7g
Red frames are regions shown in Fig.7g. n: nitrated

Supplementary Figure 24

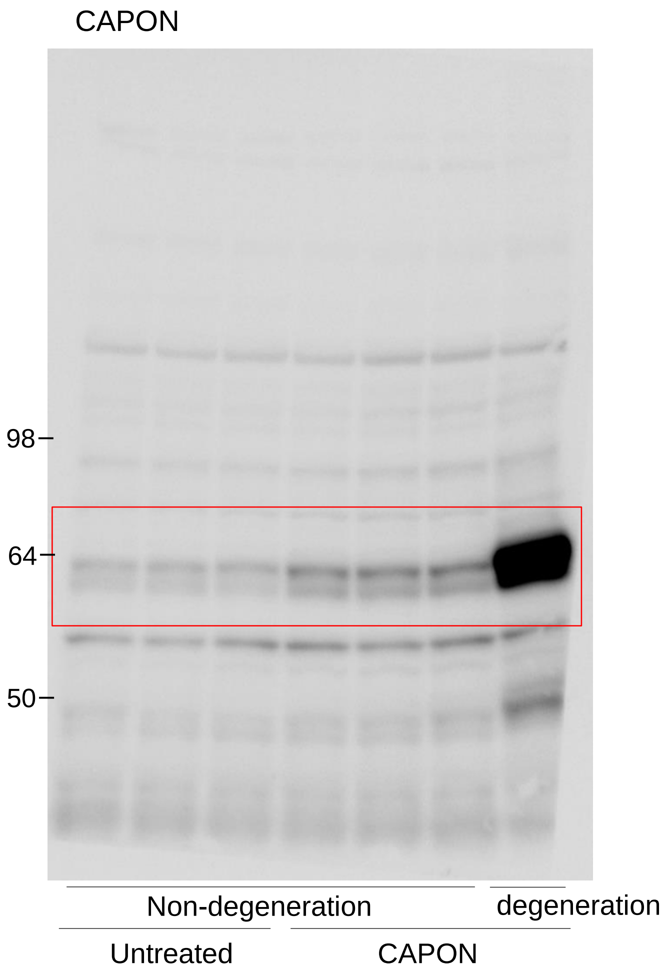

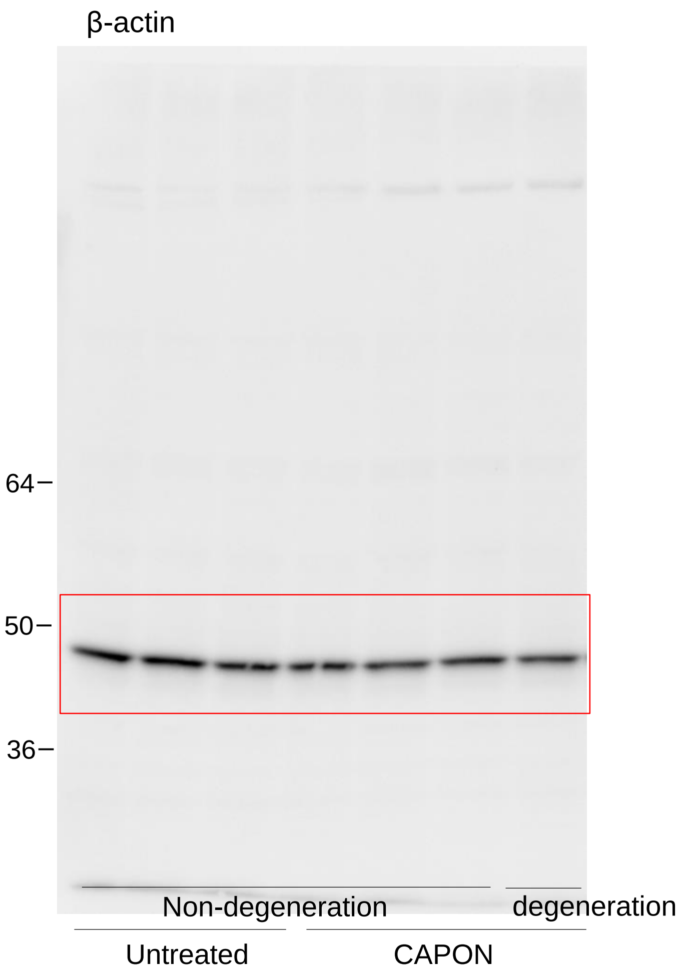

Supplementary Figure 24. The full image for Fig.8a
Red frames are regions shown in Fig.8a

Supplementary Figure 25

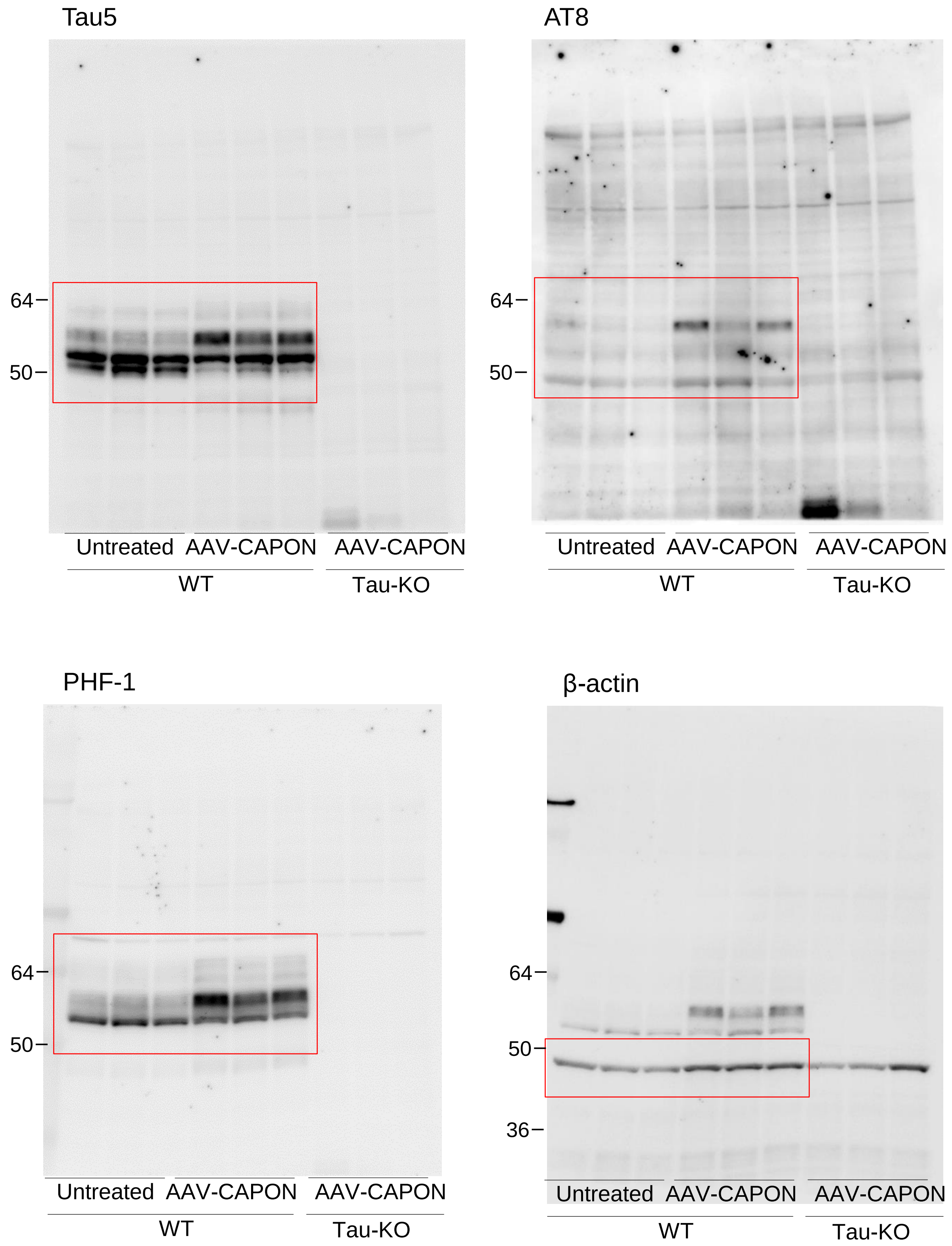

Supplementary Figure 25. The full image for Fig.8e
Red frames are regions shown in Fig. 8e

Supplementary Figure 26

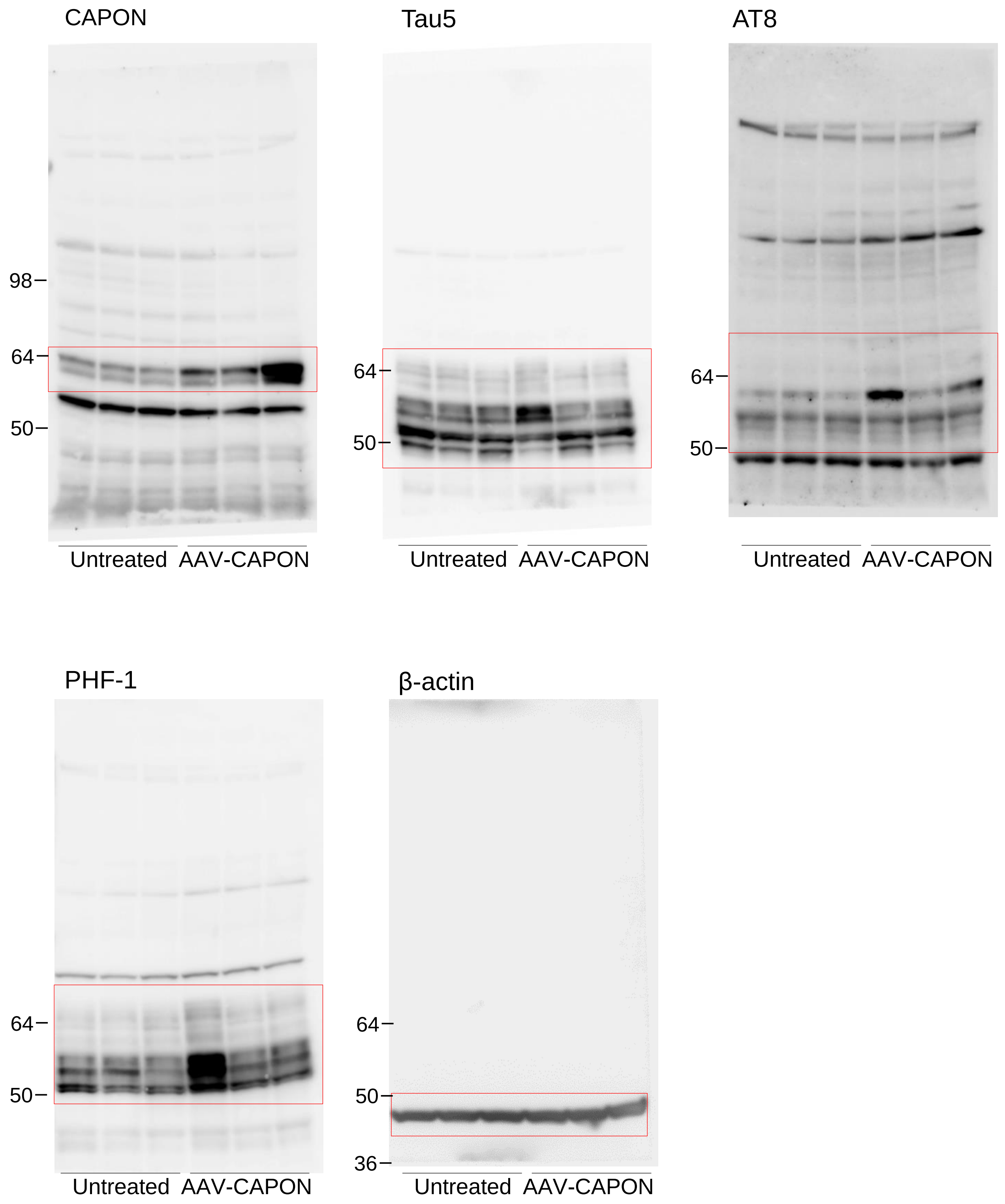

Supplementary Figure 26. The full image for Fig.8h
Red frames are regions shown in Fig. 8h

Supplementary Figure 27

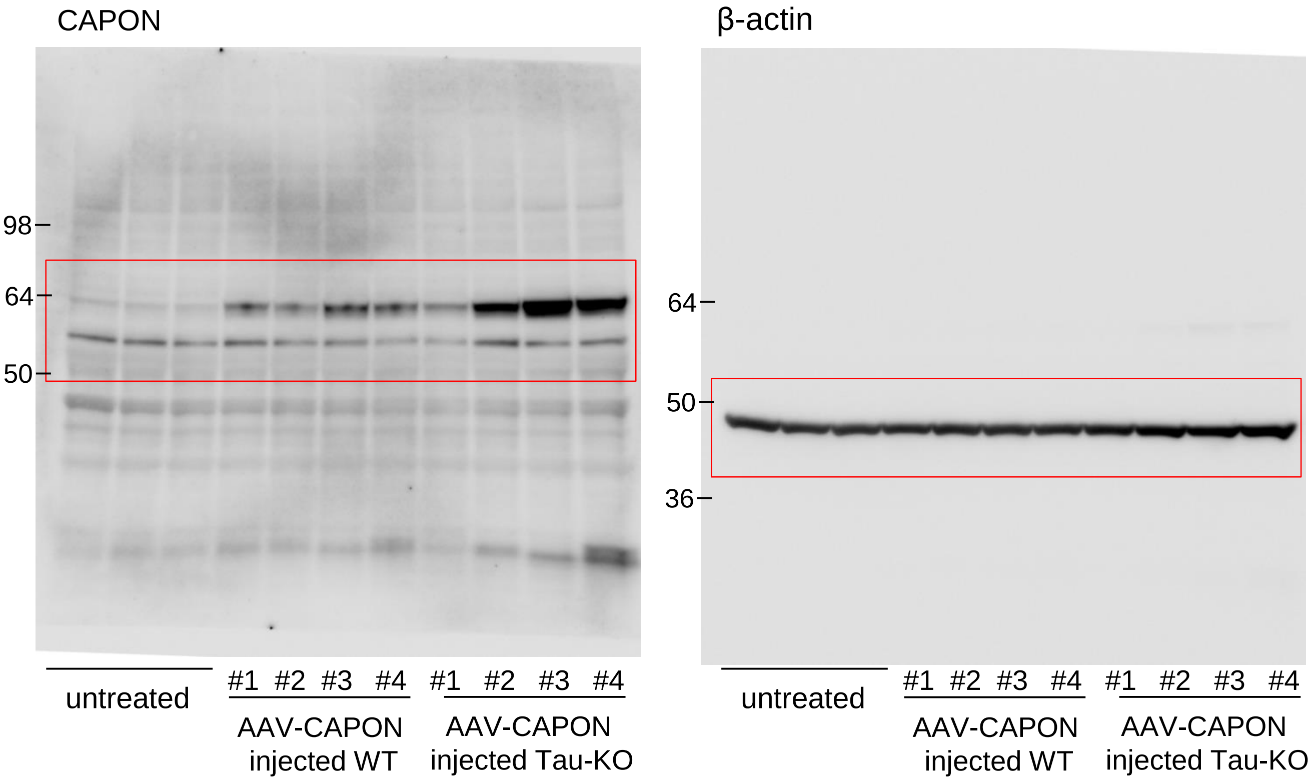

Supplementary Figure 27. The full image for Fig. 9a
Red frames are regions shown in Fig. 9a

Supplementary Figure 28

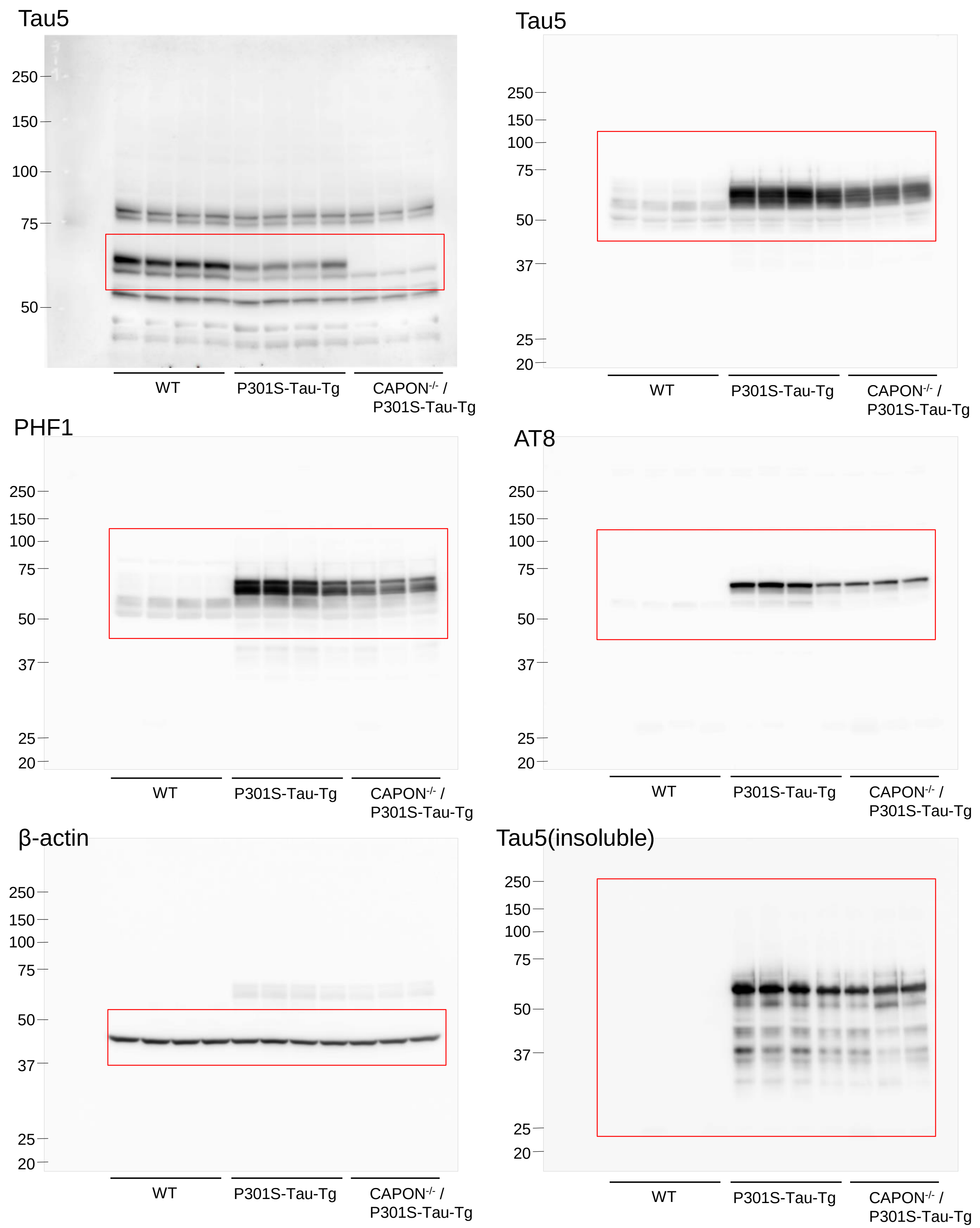

Supplementary Figure 28. The full image for Fig. 10a and b
Red frames are regions shown in Fig. 10a and b

Supplementary Figure 29

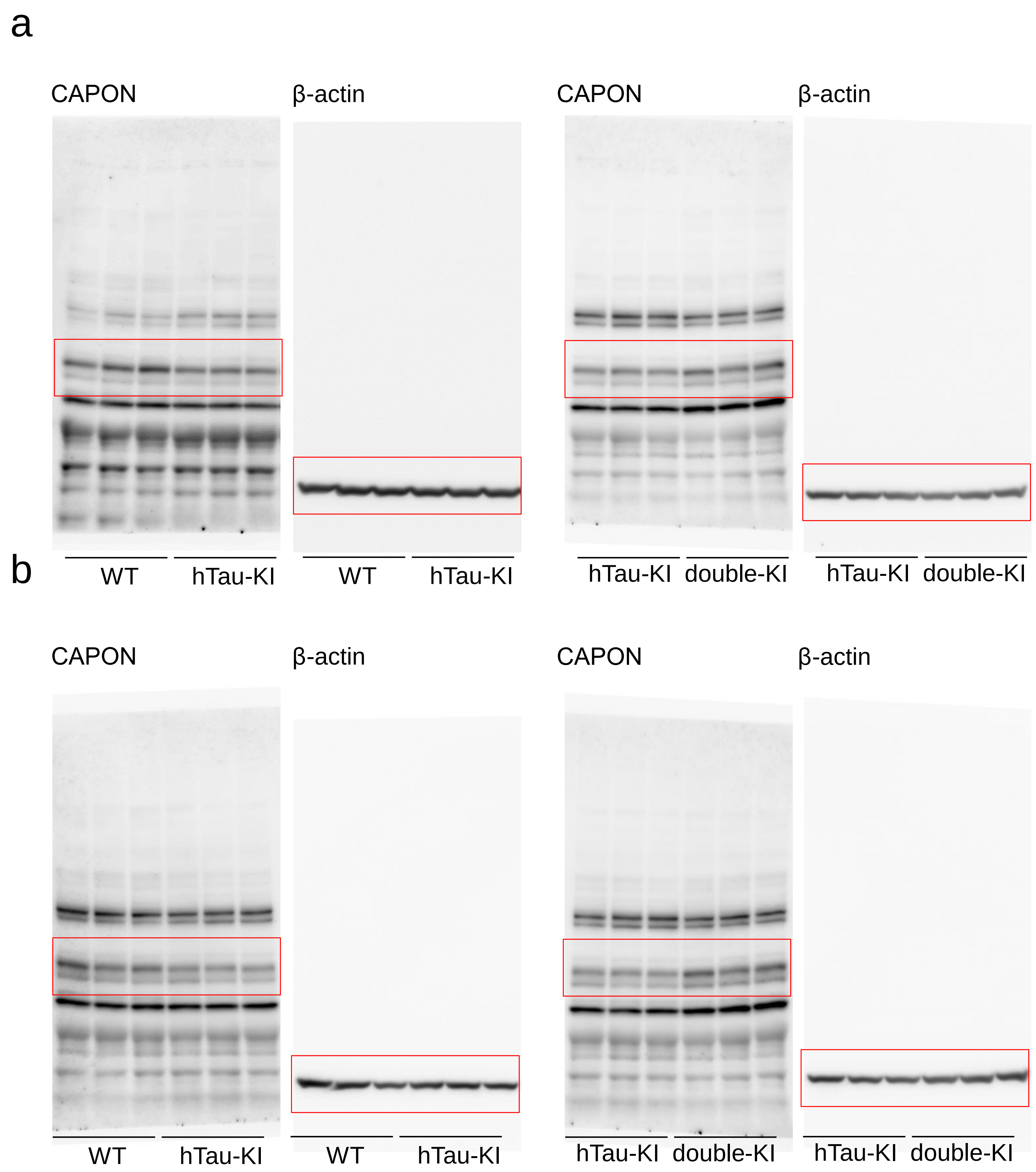

Supplementary Figure 29. The full image for Supplementary Figure 4
Red frames are regions shown in Supplementary Figure 4

Supplementary Figure 30

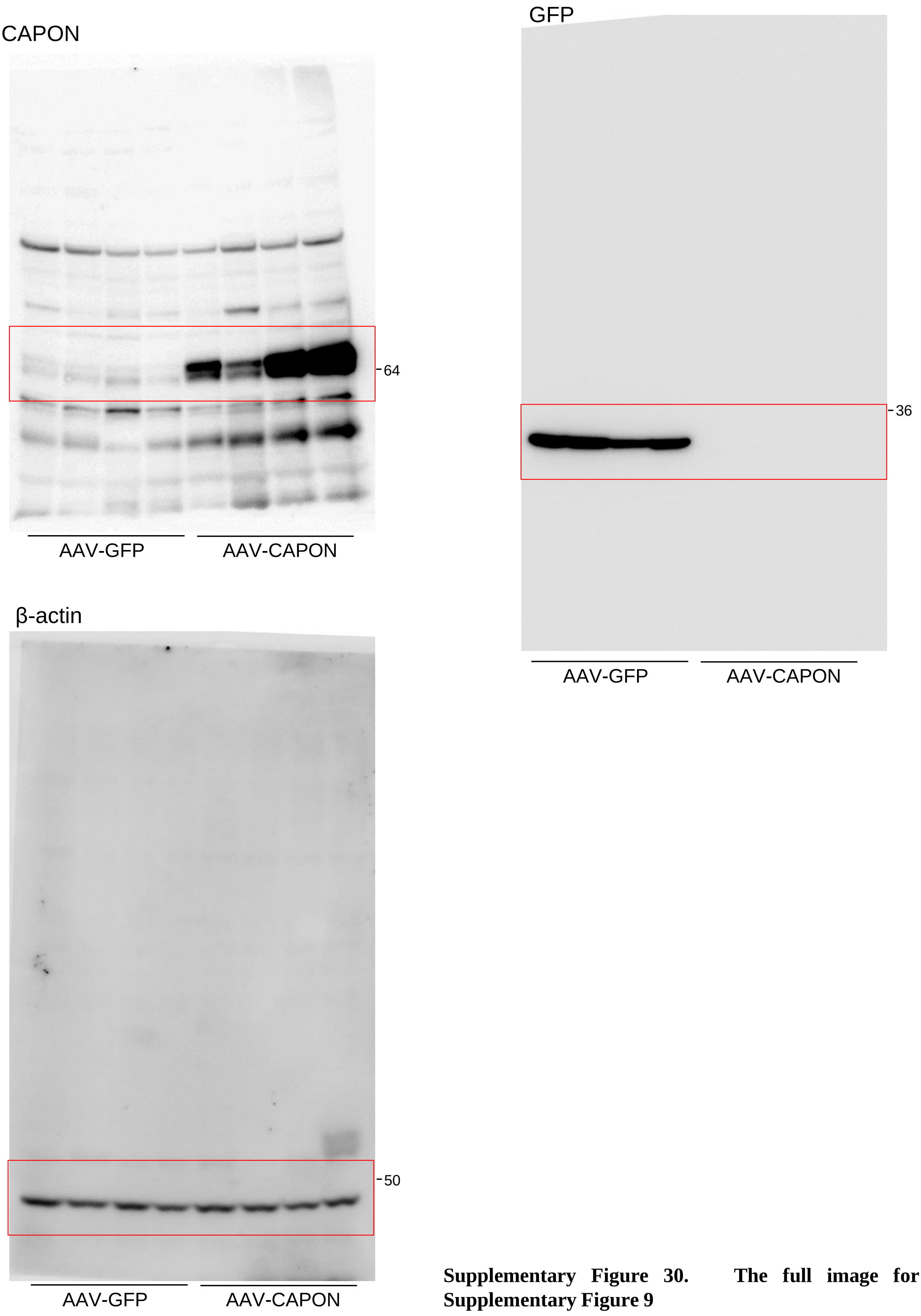

Supplementary Figure 30. The full image for Supplementary Figure 9
Red frames are regions shown in Supplementary Figure 9.

Supplementary Figure 31

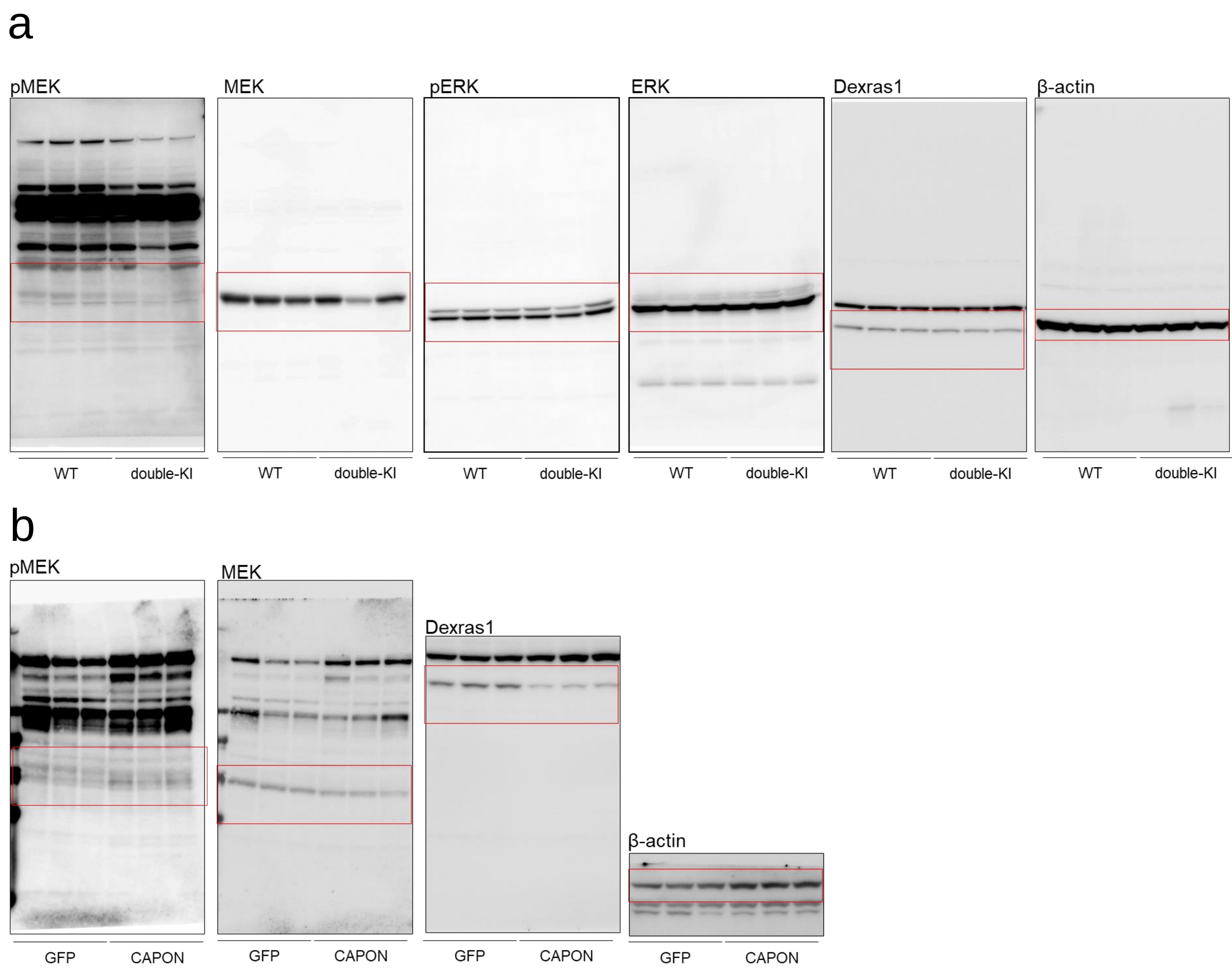

Supplementary Figure 31. The full image for Supplementary Figure 10
Red frames are regions shown in Supplementary Figure 10

Supplementary Figure 32

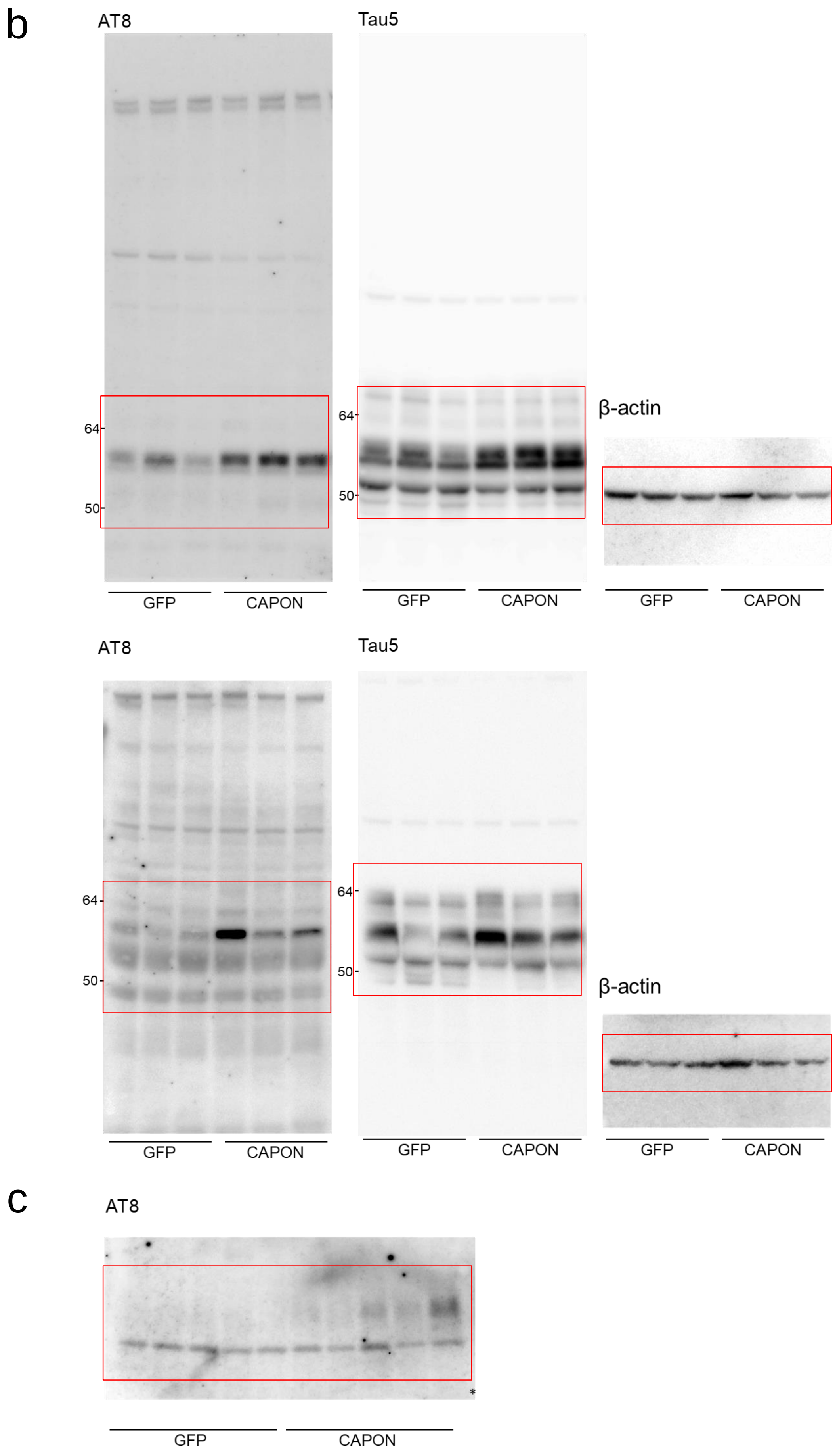

Supplementary Figure 32. The full image for Supplementary Figure 11. Red frames are regions shown in Supplementary Figure 11.

Supplementary Table 1

Supplementary Table 1. Antibodies used for western blot (WB) and immunohistochemical (IHC) analyses.

The following antibodies were used at the indicated dilutions.

	Antibody	Dilution (WB)	(IHC)
CAPON	Santa Cruz #sc374504 (clone C9)	1:2500	1:1000
CAPON	Santa Cruz #sc9138 (clone R300)	-	1:500 (Duolink)
GFP	Abcam #ab6673	1:5000	1:1000
APP/Aβ	Merck Millipore #MAB348 (clone 22c11)	1:2500	-
Aβ	Saido et al, J. Biol. Chem, 1994 (N1D)	-	1:500
Tau5 (total Tau)	Thermo #AHB0042 (Tau5)	1:2500	-
Tau13 (human Tau)	Santa Cruz #sc-21796	1:2500	-
RD3	Merck Millopore #05-803	1:1000	-
RD4	MyBiosource #MBS604301	1:1000	-
AT8 (pS202/pT205-Tau)	Innogenetics #90206 (anti PHF-TAU)	1:2500	1:200
PHF1 (pS396/pS404-Tau)	kindly provided by Peter Davis	1:2500	-
pS396-Tau	Covance #MMS-546R	1:2500	-
pS404-Tau	Abcam #ab92676	1:2500	-
pS422-Tau	Covance #PRB-524P	1:2500	-
pY18-Tau	Covance #SIG-39436-200	1:1000	-
pY29-Tau	Covance #SIG-39439-200	1:1000	-
Iba1	Wako #NCNP24	-	1:200
GFAP	Merck Millipore #MAB3402	-	1:200
nNOS	CST #4231S	1:2500	1:200
NeuN	Abcam #ab104224	-	1:500
cleaved-caspase3	CST #9661S	-	1:200
CytC	CST #4272S	-	1:200
Dexras1	Abcam #ab171370	1:5000	-
Bax	Abcam #ab32503	1:2500	-
p-ERK	CST #4695	1:1000	-
ERK	CST #9102	1:2500	-
p-MEK	CST #9121	1:2500	-
MEK	CST #9122	1:2500	-
GSDMD	Abcam #ab209845	1:1000	-
GSDME	Abcam #ab215191	1:1000	-
Olig2	Abcam #ab109186	-	1:1000
CD31	Abcam #ab28364	-	1:200
Synaptophysin	PROGEN #61412	-	1:50
VGAT	Synaptic Systems #131002	-	1:1500
MAP2	Leinoco Technologies #M119	-	1:1000
MC1	kindly provided by Peter Davis	-	1:200
β-actin	SIGMA #A5441	1:5000	-
