# Supplementary material for: Tau binding protein CAPON induces tau aggregation and neurodegeneration
Source: Nat Commun. 2019 Jun 3;10:2394. doi: 10.1038/s41467-019-10278-x (PMC6546774; doi:10.1038/s41467-019-10278-x)
Supplement: Supplementary file 3 — Description of Additional Supplementary Files [file 41467_2019_10278_MOESM3_ESM.pdf]

Description of Additional Supplementary Files

File Name: Supplementary Data 1

Description: **Tau-interacting proteins identified by tau interactome**

Tau-interacting proteins was screened by immunoprecipitation using a Flag-tag antibody and identified them by LC-MS/MS analysis. Co-immunoprecipitated proteins which were specifically identified in Wtau-Tg mice are listed.
