# Supplementary material for: Tau binding protein CAPON induces tau aggregation and neurodegeneration
Source: Nat Commun. 2019 Jun 3;10:2394. doi: 10.1038/s41467-019-10278-x (PMC6546774; doi:10.1038/s41467-019-10278-x)
Supplement: Supplementary file 5 — Reporting Summary [file 41467_2019_10278_MOESM5_ESM.pdf]

nature research

Corresponding author(s): Shoko Hashimoto, Takaomi C. Saido, Takashi Saito

Last updated by author(s): Apr 11, 2019

Reporting Summary

Nature Research wishes to improve the reproducibility of the work that we publish. This form provides structure for consistency and transparency in reporting. For further information on Nature Research policies, see Authors & Referees and the Editorial Policy Checklist.

Statistics

For all statistical analyses, confirm that the following items are present in the figure legend, table legend, main text, or Methods section.

n/a	Confirmed	
☐	☒	The exact sample size (*n*) for each experimental group/condition, given as a discrete number and unit of measurement
☐	☒	A statement on whether measurements were taken from distinct samples or whether the same sample was measured repeatedly
☐	☒	The statistical test(s) used AND whether they are one- or two-sided<br>*Only common tests should be described solely by name; describe more complex techniques in the Methods section.*
☐	☒	A description of all covariates tested
☐	☒	A description of any assumptions or corrections, such as tests of normality and adjustment for multiple comparisons
☐	☒	A full description of the statistical parameters including central tendency (e.g. means) or other basic estimates (e.g. regression coefficient) AND variation (e.g. standard deviation) or associated estimates of uncertainty (e.g. confidence intervals)
☒	☐	For null hypothesis testing, the test statistic (e.g. *F*, *t*, *r*) with confidence intervals, effect sizes, degrees of freedom and *P* value noted<br>*Give P values as exact values whenever suitable.*
☒	☐	For Bayesian analysis, information on the choice of priors and Markov chain Monte Carlo settings
☒	☐	For hierarchical and complex designs, identification of the appropriate level for tests and full reporting of outcomes
☐	☒	Estimates of effect sizes (e.g. Cohen's *d*, Pearson's *r*), indicating how they were calculated

Our web collection on statistics for biologists contains articles on many of the points above.

Software and code

Policy information about availability of computer code

Data collection	Western blot: Immuno-reactive bands were scanned with a LAS-3000mini LuminoImage analyzer (Fuji Film), and band intensity was analyzed by Image Studio Digits (LI-COR Bioscience). Immunohistochemistry and Duolink: The sections were scanned on a NanoZoomer NDP system (Hamamatsu Photonics), and signals were quantified using Definiens Tissue Studio (Definiens). MRI:The hippocampal volume of each mouse was calculated using ImageJ software.
Data analysis	All analyses were completed with Graphpad Prism7 Software (San Diego, CA, USA). Differences between groups were examined for statistical significance with Student's t-test.

For manuscripts utilizing custom algorithms or software that are central to the research but not yet described in published literature, software must be made available to editors/reviewers. We strongly encourage code deposition in a community repository (e.g. GitHub). See the Nature Research guidelines for submitting code & software for further information.

Data

Policy information about availability of data

All manuscripts must include a data availability statement. This statement should provide the following information, where applicable:

- Accession codes, unique identifiers, or web links for publicly available datasets
- A list of figures that have associated raw data
- A description of any restrictions on data availability

The datasets generated during and/or analyzed during the current study are available from the corresponding author on reasonable request.

Field-specific reporting

Please select the one below that is the best fit for your research. If you are not sure, read the appropriate sections before making your selection.

☒ Life sciences ☐ Behavioural & social sciences ☐ Ecological, evolutionary & environmental sciences

For a reference copy of the document with all sections, see nature.com/documents/nr-reporting-summary-flat.pdf

Life sciences study design

All studies must disclose on these points even when the disclosure is negative.

Sample size	The sample sized use in this study are appropriate. We carried out the experiments using 3 or more samples per group.
Data exclusions	There is no data exclusions in this study.
Replication	We confirmed reproducibility by multiple independent data collections.
Randomization	Randomization is not relevant to our study. We did not chose samples from among many, but used all samples for experiments.
Blinding	The investigators were not blinded during data collection, because first author carried out both designing of experiments and quantitative assessments. However, two or more persons agree with the interpretation of experimental data.

Reporting for specific materials, systems and methods

We require information from authors about some types of materials, experimental systems and methods used in many studies. Here, indicate whether each material, system or method listed is relevant to your study. If you are not sure if a list item applies to your research, read the appropriate section before selecting a response.

Materials & experimental systems

n/a	Involved in the study	
☐	☒	Antibodies
☒	☐	Eukaryotic cell lines
☒	☐	Palaeontology
☐	☒	Animals and other organisms
☒	☐	Human research participants
☒	☐	Clinical data

Methods

n/a	Involved in the study	
☒	☐	ChIP-seq
☒	☐	Flow cytometry
☐	☒	MRI-based neuroimaging

Antibodies

Antibodies used

CAPON (WB, IHC): Santa Cruz #sc374504(clone C9)
CAPON(Duolink): Santa Cruz #sc9138 (clone R300)
GFP: Abcam #ab6673
APP/Aβ: Merck Millipore #MAB348 (clone 22c11) Aβ: Saido et al, J. Biol. Chem, 1994 (N1D)
Tau5 (total Tau): Thermo #AHB0042 (Tau5)
AT8 (pS202/pT205-Tau): Innogenetics #90206 (anti PHF-TAU)
PHF1 (pS396/pS404-Tau): kindly provided by Peter Davis
pS396-Tau: Covance #MMS-546R
pS404-Tau: Abcam #ab92676
pS422-Tau: Covance #PRB-524P
pY18-Tau: Covance #SIG-39436-200
pY29-Tau: Covance #SIG-39439-200
Iba1: Wako #NCNP24
GFAP: Merck Millipore #MAB3402
nNOS: CST #4231S
NeuN: Abcam #ab104224
cleaved-caspase3: CST #9661S
CytC: CST #4272S
Dexras1: Abcam #ab171370
Bax: Abcam #ab32503
p-ERK: CST #4695
ERK: CST #9102
p-MEK: CST #9121
MEK: CST #9122
β-actin: SIGMA #A5443

nature research | reporting summary October 2018
Tau13(human Tau):Santa Cruz #sc-21796
RD3:Merck Millopore #05-803
RD4:MyBiosource #MBS604301
GSDMD:Abcam #ab209845
GSDME:Abcam #ab215191
Olig2:Abcam #ab109186
CD31: Abcam #ab28364
Synaptophysin:PROGEN #61412
VGAT:Synaptic Systems #131002
MAP2:Leinoco Technologies #M119
MC1:kindly provided by Peter Davis

Validation

CAPON: WB (1:2500), IHC (1:1000)
CAPON: Duolink (1:500)
GFP: WB (1:5000), IHC (1:1000)
APP/Aβ: IHC (1:2500)
Aβ(N1D): IHC (1:500)
Tau5 (total Tau)(Tau5): WB (1:2500)
AT8: WB (1:2500), IHC (1:200)
PHF1: WB (1:2500)
pS396-Tau: WB (1:2500)
pS404-Tau: WB (1:2500)
pS422-Tau: WB (1:2500)
pY18-Tau: WB (1:1000)
pY29-Tau: WB (1:1000)
Iba1: IHC (1:200)
GFAP: IHC (1:200)
nNOS: WB (1:2500), IHC (1:200)
NeuN: IHC (1:500)
cleaved-caspase3: IHC (1:200)
CytC: IHC (1:200), WB (1:1000)
Dexras1: WB (1:5000)
Bax: WB (1:2500)
p-ERK: WB (1:1000)
ERK: WB (1:2500)
p-MEK: WB (1:2500)
MEK: WB (1:2500)
β-actin: WB (1:5000)
Tau13 (human Tau): WB (1:2500)
RD3 Merck Millopore: WB (1:1000)
RD4 MyBiosource: WB (1:1000)
GSDMD: WB (1:1000)
GSDME :WB (1:1000)
Olig2: IHC (1:1000)
CD31: IHC (1:200)
Synaptophysin: IHC (1:50)
VGAT Synaptic Systems: IHC (1:1500)
MAP2: IHC (1:1000)
MC1: IHC (1:200)

Animals and other organisms

Policy information about studies involving animals; ARRIVE guidelines recommended for reporting animal research

Laboratory animals

All animal experiments were conducted in accordance with the guidelines of the RIKEN Center for Brain Science. All strains were maintained on a C57BL/6 background. Sex and age of mice used in each experiments were described in figure legends.

Wild animals

No wild animals were used

Field-collected samples

No field-collected samples were used

Ethics oversight

All animal experiments were conducted in accordance with the guidelines of the RIKEN Center for Brain Science.

Note that full information on the approval of the study protocol must also be provided in the manuscript.

Magnetic resonance imaging

Experimental design

Design type

Resting state

Design specifications | n/a

Behavioral performance measures | n/a

Acquisition

Imaging type(s)	Structural MRI
Field strength	9.4 tesla
Sequence & imaging parameters	T2W scans were performed with the following parameter settings: TR (repetition time) = 4342.2 ms, TE (echo time) = 53.8ms, matrix dimensions = 256 x 256, flip angle = 180 degrees, field of view = 1.8cm x 1.8cm.
Area of acquisition	Whole brain

Diffusion MRI ☐ Used ☒ Not used

Preprocessing

Preprocessing software	Scans (2D TurboRAGE) of the whole brain were performed with a vertical-bore 9.4 T Bruker AVANCE 400WB imaging spectrometer with a 250 mTm-1 actively shielded imaging gradient insert (Bruker BioSpin) controlled by Paravision 5.1 software (Bruker Biospin).
Normalization	n/a
Normalization template	n/a
Noise and artifact removal	n/a
Volume censoring	n/a

Statistical modeling & inference

Model type and settings	n/a
Effect(s) tested	n/a

Specify type of analysis: ☐ Whole brain ☐ ROI-based ☐ Both

Statistic type for inference (See Eklund et al. 2016)	n/a
Correction	n/a

Models & analysis

n/a	Involved in the study
☒	☐ Functional and/or effective connectivity
☒	☐ Graph analysis
☒	☐ Multivariate modeling or predictive analysis
